# High-resolution mapping of embryonic genome activation unveils a decoupling of transcription activation from precocious H3K4me3 removal

**DOI:** 10.1126/sciadv.aec9545

**Published:** 2026-08-07

**Authors:** Jasmina Al-Mousawi, Luca Michetti, Laura Castaldi, Na Liu, Laura Villacorta, Vladimir Benes, Ana Boskovic

**Affiliations:** ^1^ European Molecular Biology Laboratory (EMBL) – Epigenetics and Neurobiology Unit, Via E. Ramarini 32, Monterotondo, 00015 Rome, Italy.; ^2^ Sapienza University of Rome, PhD Program in Genetics and Molecular Biology, Piazzale Aldo Moro 5, 00185 Rome, Italy.; ^3^ European Molecular Biology Laboratory (EMBL), Genomics Core Facility, Meyerhofstraße 1, 69117 Heidelberg, Germany.

## Abstract

The lack of temporal resolution in transcriptomic data during mammalian embryonic genome activation (EGA) has precluded the comprehensive understanding of the functional relationships between the various gene regulatory mechanisms governing this process. Here, we finely dissect the transcriptional dynamics of mouse EGA using precision in vitro fertilization (IVF) coupled with single-embryo RNA sequencing. Our highly temporally resolved dataset uncovers an extensive, step-wise remodeling of the embryonic messenger RNA landscape, affecting ∼30% of the total detectable transcripts over a 9-hour time frame. We capture the gradual shift from maternal to embryonic messenger RNAs, successfully identify ribosome biogenesis and translation as hallmarks of EGA, and find previously unidentified gene expression dynamics. We further uncover a set of eight histone demethylating enzymes among the earliest up-regulated EGA genes and leverage our precision-IVF to dissect the transcriptional versus developmental impact of histone H3 lysine-4 trimethylation (H3K4me3) remodeling after fertilization. Our results indicate that precocious removal of H3K4me3 from embryonic chromatin only modestly affects embryonic transcription without perturbing EGA timing, arguing against a major instructive role of precocious remodeling of maternally inherited H3K4me3 after fertilization on genome activation. High-resolution transcriptome mapping coupled with functional perturbations allows us to distinguish direct gene expression effects from general impacts on developmental timing, opening avenues for further quantitative characterization of the impact of epigenome remodeling on embryonic transcription.

## Teaser

Precision-IVF enables high-resolution characterization of mouse embryonic genome activation.

## INTRODUCTION

Embryonic genome activation (EGA) is the onset of embryonic transcription after fertilization, following a state of transcriptional quiescence inherited from the gametes. In the mouse, EGA occurs in two waves: a minor zygotic wave characterized by low-level transcription of genes that are both aberrantly spliced and polyadenylated and a major wave of productive transcription during the 2-cell stage (*1*). Major EGA, referred to as EGA henceforth, is characterized by transcriptional up-regulation of thousands of genes and transposable elements (TEs), occurring in parallel with the depletion and degradation of maternally deposited transcripts (*2*, *3*). Concomitantly with EGA, pervasive remodeling of the epigenome takes place, including, but not limited to, the clearance and reestablishment of histone posttranslational modifications (PTMs), erasure of DNA methylation patterns, changes in chromatin accessibility, and three-dimensional nuclear organization [reviewed in (*4*–*6*)]. These co-occurring global changes to the epigenome pose a challenge in assessing their quantitative impact and relative contribution, both functional and temporal, to EGA regulation. Precise temporal understanding of epigenetic changes at EGA is critical for understanding their functional interconnections.

In many well-studied EGA model systems, such as *Drosophila melanogaster* or *Danio rerio*, transcriptomic and chromatin features during EGA have been mapped at temporal resolutions in the order of minutes (*7*–*12*), facilitated by precise fertilization timing, highly synchronous cleavages, and availability of biological material. Data from mammalian embryos currently lack on these considerations: Fertilization timing is imprecise and is on the scale of hours, except when embryos are derived through intracytoplasmic sperm injections (ICSIs) (*13*, *14*); cleavages are asynchronous (*15*, *16*); and the availability of experimental material is prohibitively low. In the mouse, this has traditionally led to the digital sampling and profiling of early versus late 2-cell stage embryos, corresponding to pre-EGA and post-EGA states, with estimated fertilization time based on injection of ovulation-inducing hormone human chorionic gonadotropin (hCG) (*17*–*20*). While important strides in understanding the mechanisms of mammalian EGA have been made using this approach, considerable limitations remain. For instance, intermediate states of EGA remain unexplored, obscuring potential critical regulators of the process. Furthermore, asynchrony in developmental timing due to differences in staging can result in the appearance or masking of transcriptional phenotypes.

Here, we tackle these limitations by combining precision embryo staging and single-embryo transcriptomics to obtain a high-resolution transcriptomic profile of mouse embryos undergoing EGA. We ask how the expression of genes and TEs is coordinated during EGA and whether regulatory pathways or expression dynamics associated with this phenomenon can be uncovered. We find that 4871 genes are up-regulated between pre- and post-EGA, while 2266 genes decrease concomitantly, during a 9 -hour period of the 2-cell stage. Our high-resolution dataset reveals that specific gene sets exhibit distinct temporal expression patterns, ranging from progressive increase and depletion to highly dynamic and time point specific.

We identify *Kdm5b*, a demethylase of histone H3 lysine 4 trimethylation (H3K4me3), as one of the earliest upregulated genes during EGA. While H3K4me3 is canonically associated with active promoters, in transcriptionally quiescent oocytes, H3K4me3 spans both distal regions and silent promoters (*21*). The establishment of these broad, noncanonical H3K4me3 (ncH3K4me3) domains occurs concomitantly with transcriptional silencing during the nonsurrounded nucleus (NSN) to the surrounded nucleolus (SN) transition (*21*–*24*). Overexpression of Kdm5b in SN oocytes was reported to induce ectopic transcription, pointing to a role of ncH3K4me3 in promoting genome silencing (*22*, *24*). Kdm5b-mediated clearance of ncH3K4me3 from the 2-cell stage onward was shown to be necessary for blastocyst development (*21*). However, it is unclear at which point during EGA ncH3K4me3 removal occurs and whether the transcription of specific EGA genes depends on it. Thus, we leveraged our precision in vitro fertilization (precision-IVF) to measure the effect of precocious removal of H3K4me3 on EGA, in terms of its timing and gene expression dynamics. We find that the removal of H3K4me3 before EGA only modestly affects the embryonic transcriptome at the 2-cell stage, without changing EGA timing or compromising preimplantation development. Our findings represent the first temporally resolved assessment of the quantitative impact of a histone PTM on EGA, considering both gene expression and developmental timing. We underscore the importance of delineating these two phenotypic readouts when studying the relative contributions and temporal sequence of epigenetic events during early mammalian development.

## RESULTS

To gain precise temporal insight into the transcriptomic changes during mouse EGA, we first sought to establish an experimental pipeline to measure mRNA content of highly synchronous individual embryos at specific intervals during the 2-cell stage. In particular, we were interested in capturing intermediate states of EGA, as these might lend biological insight into EGA regulation and simultaneously report on the robustness of EGA progression across biological replicates.

**Fig. 1.**
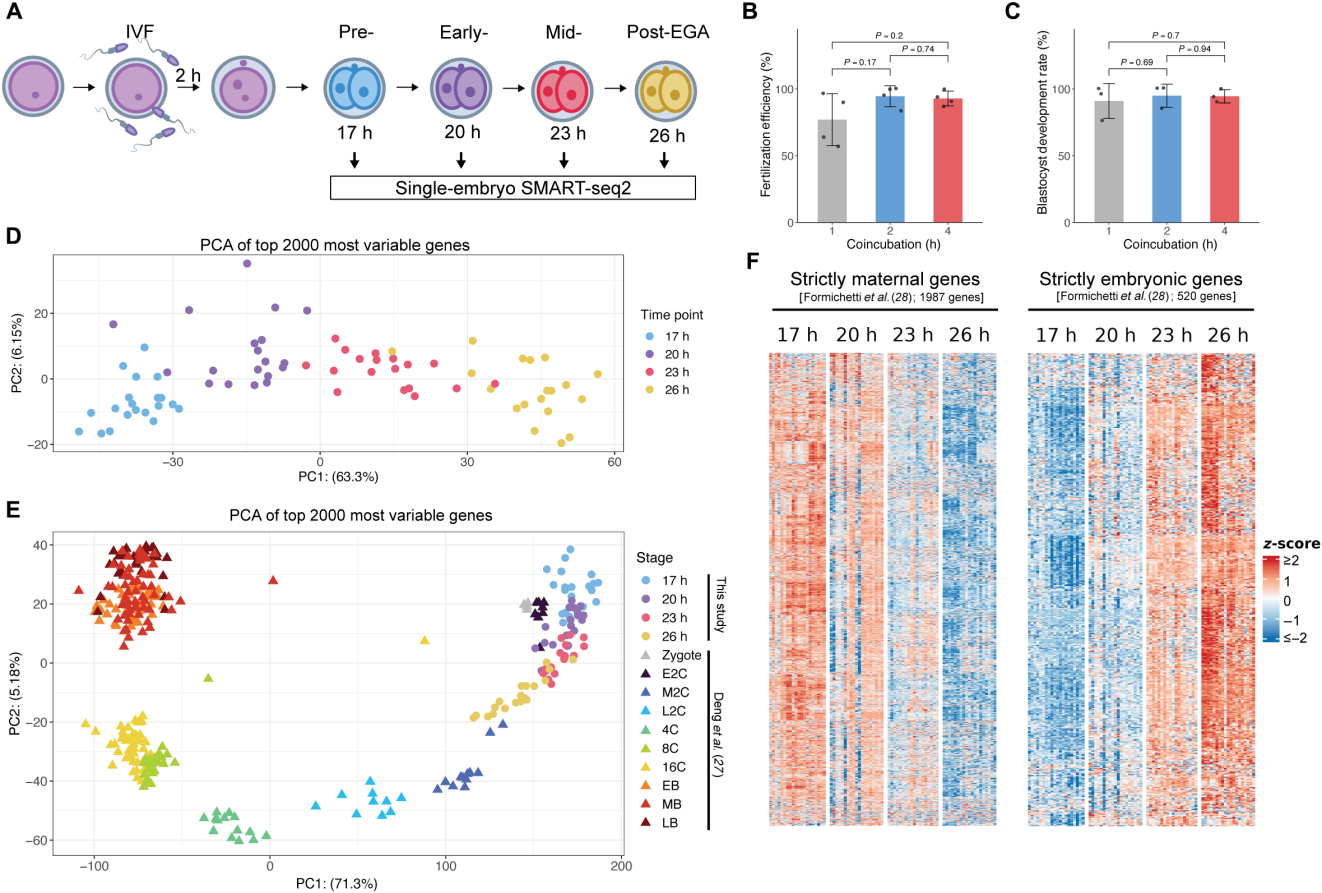
High-resolution mapping of the mouse transcriptome during EGA. (**A**) Schematic of the experimental setup. The IVF protocol has been modified to shorten the coincubation of oocytes and sperm to narrow down the fertilization window. Collection time points post-IVF are indicated. h, hours. (**B**) Fertilization efficiency after the indicated sperm and oocyte coincubation times. The fertilization efficiency is measured as the fraction of oocytes that were fertilized and developed to the 2-cell stage. *N* = 4 IVF experiments, *n* = 273 oocytes. The groups were compared using Student’s *t* tests. (**C**) Blastocyst development rate measured as the fraction of 2-cell stage embryos that developed into healthy blastocysts. *N* = 3 IVF experiments, *n* = 212 embryos. The groups were compared using Student’s *t* tests. (**D**) Principal components analysis (PCA) of single embryos sequenced using SMART-seq2. The panel shows the top 2000 most variable genes after DESeq2 count normalization and variance stabilizing transformation. *N* = 3 IVF experiments, *n* = 76 embryos. (**E**) PCA generated using the same strategy as in (D), integrating our data with public data downloaded from (*27*). E2C, early–2-cell; M2C, mid–2-cell; L2C, late–2-cell; 4C, 4-cell, 8C, eight-cell; 16C, 16-cell; EB, early blastocyst; MB, mid-blastocyst; LB, late blastocyst. (**F**) Heatmaps of strictly maternal genes (left) (1987 genes displayed) and strictly embryonic genes (right) (520 genes displayed) visualizing *z*-scores of log(CPM + 1) counts. The gene lists used were from (*28*).

To obtain temporal precision in fertilization timing, we generated embryos using IVF and reduced the length of insemination from the standard 4 to 1 or 2 hours. Across four biological replicates, we observed a robust fertilization rate (ranging from 86.7 to 100%, 182 oocytes analyzed in total) when oocytes were incubated with spermatozoa for 2 or 4 hours (Fig. 1, A and B). In two of the four biological replicates, 1-hour coincubation led to over 90% fertilization efficiency, while the fertilization efficiency in the remaining two replicates was lower (57.1 and 64%, 91 oocytes in total) (Fig. 1B). When allowed to progress through preimplantation development, all experimental groups of embryos reached the blastocyst stage at embryonic day 4 at comparable and high rates (91, 95, and 94.4% corresponding to 1-, 2-, and 4-hour coincubation groups, respectively, 212 embryos analyzed in total) (Fig. 1C). Therefore, we opted to shorten the traditional IVF protocol to a 2-hour incubation period, as it consistently resulted in robust fertilization rates without compromising subsequent development. We refer to this optimized procedure as precision-IVF henceforth.

To test whether this method is precise and sensitive enough to reveal gradual transcriptomic changes throughout EGA, we collected individual, morphologically indistinguishable 2-cell stage embryos at the following time points after IVF: 17, 20, 23, and 26 hours, corresponding to pre-, early-, mid-, and post-EGA, respectively (Fig. 1A) (*2*). The transcriptomes of single embryos were profiled using the SMART-seq2 protocol (*25*, *26*). Principal components analysis (PCA) of 2-cell stage embryo transcriptomes collected during the EGA time course revealed that they separated along principal component 1 (PC1), which corresponds to developmental time. Most of the variance in the data (63.3%) is explained along PC1 (Fig. 1D), indicating that the transcriptome remodeling proceeds in a largely stereotypical and robust manner. In contrast, PC2, reporting on 6.15% of the variance, corresponded to interembryo variability. Hierarchical clustering showed that the samples clustered by time point and identified two main clusters: one representing the pre- and early-EGA transcriptomes (17 and 20 hours) and the other representing the mid-to-late-EGA transcriptomes (23 and 26 hours) (fig. S1A). Integration with previously published single-cell RNA sequencing (RNA-seq) data encompassing preimplantation development from the zygote to the blastocyst stage (*27*) showed that our samples resolve the gap between the early–2-cell and mid–2-cell stage and thus accurately capture the dynamic reshaping of the transcriptome during the onset of EGA, with high temporal resolution (Fig. 1E and fig. S1B). In addition, we compared the mean normalized DESeq2 counts across biological replicates in a pairwise manner at different collection points (fig. S1C). We observed high correlation between biological replicates at all analyzed time points, with squared Pearson’s correlation coefficients ranging from 0.81 to 0.92, attesting to the experimental reproducibility and robustness of the method. Last, we examined the expression dynamics of strictly maternal and strictly embryonic genes based on previously curated gene lists (*28*) and observed a gradual depletion of maternal transcripts and concomitant up-regulation of embryonic ones during the 9-hour collection window (Fig. 1F), demonstrating that EGA proceeded without hindrance.

To ensure that our SMART-seq2 approach captures maternal transcripts, which undergo deadenylation before and during EGA (*29*–*31*), we generated an additional EGA time course dataset where we compared the transcriptomes of individual embryos with or without RNA in vitro polyadenylation (henceforth referred to as PolyA+ and PolyA−, respectively). Briefly, the lysates from individual embryos were split, and in vitro polyadenylation was performed on half of the sample to capture total RNA. SMART-seq2 libraries were built individually for both fractions (fig. S2A). PCA showed that the temporal dynamics were maintained under both conditions (fig. S2B). As expected, we observed an enrichment of naturally nonpolyadenylated transcripts in PolyA+ samples, including histone mRNAs and small noncoding RNAs (fig. S2, C to F, and table S5). This was reflected in the accompanying Gene Ontology (GO) analysis, which showed that structural components of chromatin were enriched in PolyA+ samples (fig. S2, G to J). The dynamic behavior of maternal genes in both fractions (fig. S2, K and L) closely mirrored that shown in Fig. 1F, indicating that our original approach accurately captures the depletion of maternal transcripts. Thus, all downstream analyses were performed on our original SMART-seq2 dataset.

**Fig. 2.**
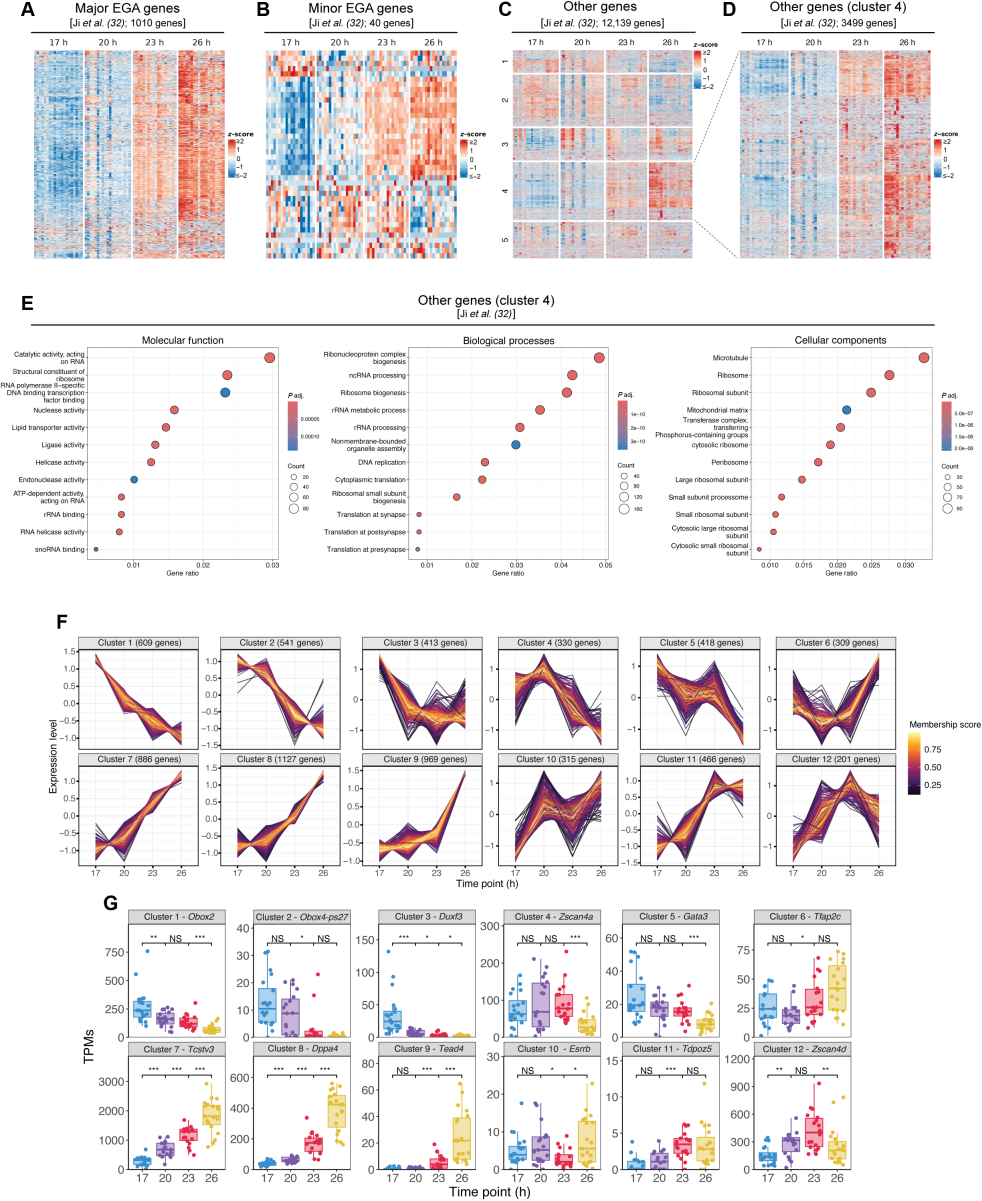
Characterizing EGA transcriptome dynamics. (**A** and **B**) Heatmaps of previously published major EGA genes (A) (1010 genes visualized) and minor EGA genes (B) (40 genes visualized), visualizing *z*-scores of log(CPM + 1) counts. The gene lists used were from (*32*). h, hours. (**C**) Heatmap of other (non-EGA) genes reported in (*32*). The heatmap has been generated as in (A) and (B), with the difference that *k*-means clustering using five clusters has been applied to the rows. A total of 12,139 genes are displayed. (**D**) Heatmap of cluster 4 from (C) generated using the same strategy as (A) and (B). A total of 3499 genes are displayed. (**E**) GO of cluster 4 from (C) and (D) generated using ClusterProfiler. Top 12 categories are visualized. snoRNA, small nucleolar RNA; ncRNA, noncoding RNA. (**F**) Fuzzy *c*-means clustering of all dynamic genes across all comparisons, defined as genes with DESeq2 parameters |log_2_(FC)| > 1 and *P* adj. < 0.05 for all comparisons. Genes with a TPM sum of medians of <5 were filtered out. Clustering was calculated using gene expression medians and the mfuzz package, with parameters *c* = 12 and *m* = 1.8. (**G**) TPM levels of select genes from clusters presented in (F). The groups are compared using Wilcoxon rank sum tests. The significance is indicated as *P < 0.05, **P < 0.01, ***P < 0.001, and NS (not significant).

Next, we tested the expression dynamics of previously annotated gene sets associated with major EGA, minor EGA, and the remaining transcriptome (“other” genes) (Fig. 2, A to C) (*32*). We successfully captured the progressive up-regulation of major EGA genes (Fig. 2A). We found that up to half of the annotated minor EGA genes displayed major EGA–like expression patterns (Fig. 2B), characterized by steady up-regulation during the 9-hour time course. Upon *k*-means clustering of genes annotated as other (Fig. 2C), we found an uncharacterized set of major EGA–like genes (other cluster 4) (Fig. 2D), which was enriched for GO terms related to RNA processing, translation, and transcriptional regulation (Fig. 2E), consistent with reported functions for EGA genes (*3*). Collectively, these results show that by coupling our precision-IVF protocol with single-embryo RNA-seq, we can capture both end-point and intermediate transcriptional states of mouse EGA.

We next explored the global transcriptome reshaping during EGA, taking an unbiased approach. First, we defined all differentially expressed genes (DEGs) using DESeq2 (*33*) {|log_2_[fold change (FC)]| > 1 and adjusted *P* value (*P* adj.) < 0.05 for all comparisons} across all collected time points and examined their behavior throughout EGA. We calculated median transcript per million (TPM) values for each gene per time point, filtering out lowly expressed genes (sum of medians < 5). We defined 12 gene clusters using fuzzy *c*-means clustering (see Materials and Methods), with each gene assigned to a single cluster that best represented its expression patterns. The clusters contained between 201 and 1127 genes, with distinct temporal behaviors (Fig. 2F, table S1). On the basis of their expression, these can be broadly categorized into three classes: maternal-like (clusters 1 to 4), characterized by time-dependent depletion or degradation; complex (clusters 5, 6, 10, and 12), showing transient up- or down-regulation at different time points; and embryonic-like (clusters 7 to 9 and 11), whose expression levels increase as development progresses. When analyzing genes associated with individual clusters (Fig. 2G), we found examples of previously reported EGA regulators, such as *Obox2* and *Zscan4a* in clusters 1 and 4, respectively, characterized by maternal-like temporal expression patterns. This is consistent with their reported roles as early-acting factors contributing to the transcriptional reactivation of the embryonic genome (*32*, *34*). Conversely, *Tfap2c* and *Tead4*, found in clusters 6 and 9, respectively, are up-regulated later during EGA, corroborating their ascribed functions in lineage allocation during later embryogenesis (*35*, *36*). Together, our unbiased approach allows for the exploration of the temporal dynamics of both known and putative novel regulators of early embryonic gene expression.

**Fig. 3.**
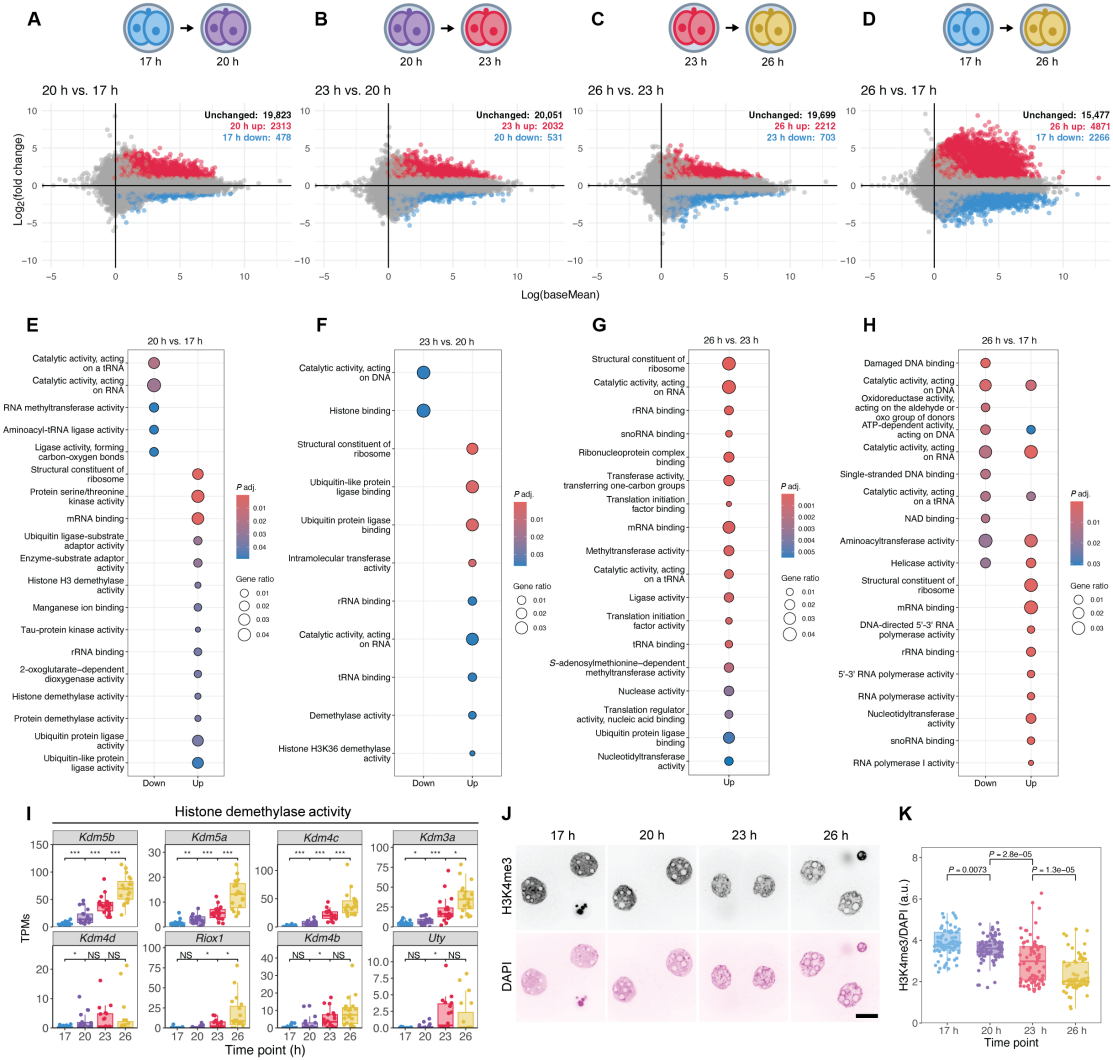
Early EGA is associated with histone demethylation. (**A** to **D**) Gene expression differences between the indicated time points, determined by DESeq2 analysis using |log_2_(FC)| > 1 and *P* adj. < 0.05 parameters for all comparisons. The baseMean value is the mean expression level of each gene calculated on the basis of DESeq2-normalized counts across all samples in the dataset. h, hours. (**E** to **H**) GO analysis of the DEGs from (A) to (D) using ClusterProfiler. The molecular function category is displayed. Up to 18 most significant terms are shown. (**I**) TPM levels of genes contributing to the “histone demethylase activity” GO term in (E) The groups are compared using Wilcoxon rank sum tests. The significance is indicated as *P < 0.05, **P < 0.01, ***P < 0.001, and NS. (**J**) Representative immunofluorescence images of H3K4me3 during EGA. Single z-slices are displayed. Scale bar, 20 μm. (**K**) Quantification of the data presented in (J), measured as nuclear H3K4me3 intensity normalized to the nuclear DAPI signal. The quantifications were done on single slices, where the nucleus was most visible. *N* = 3 IVF experiments, *n* = 312 blastomeres quantified. The groups have been compared using Student’s *t* tests. a.u., arbitrary units.

We next sought to further investigate the stepwise transcriptomic remodeling taking place during discrete stages of EGA. We performed differential gene expression analysis across adjacent time points, as well as the two end collection times (17 and 26 hours) (Fig. 3, A to D). We find that, when comparing end-point data, 31.6% of all detectable transcripts change expression levels, comprising 4871 and 2266 significantly up- and down-regulated transcripts, respectively (Fig. 3D and table S3). The GO analysis of DEGs across the two end points revealed a depletion of genes associated with single-stranded and damaged DNA binding and an up-regulation of GO terms related to transcription (DNA-directed 5′-3′ RNA-polymerase activity, mRNA binding, and RNA–polymerase I activity) and translation [structural constituent of the ribosome and ribosomal RNA (rRNA) binding] (Fig. 3H). Further examining DEGs between sequential collection times, we detected between 2032 and 2313 up-regulated genes and between 478 and 703 down-regulated ones for each pairwise comparison (Fig. 3, A to C, and table S3). The earliest up-regulated genes (from 17 to 20 hours) relate to ubiquitin protein ligase activity, mRNA binding, protein kinase activity, and histone demethylation (Fig. 3E). At the same time, tRNA-related terms (aminoacyl-tRNA ligase and catalytic activity on tRNA) are down-regulated, pointing toward a decrease in tRNA charging (Fig. 3E). Already at the following time points (20 to 23 hours), we no longer observe kinase activity among the top up-regulated terms but rather detect a down-regulation of genes involved in histone binding and catalytic activity on DNA (Fig. 3F). Gene sets belonging to rRNA and tRNA binding and ribosome biogenesis appear to be up-regulated from 20 to 23 hours (Fig. 3F) and dominate the GO term signature at the 23- to 26-hour transition (Fig. 3G).

We then examined the dynamics of transcripts mapping to TEs (fig. S3, A to D). Between 17 and 23 hours, we note a decrease of multiple LINE-1 families and increased expression of long terminal repeat (LTR) retrotransposons, including MT2s, ORR1s, and IAPs (fig. S3E). EGA has long been associated with the expression of murine endogenous retrovirus primed by tRNA leucine (MERVL) and its LTRs MT2_Mm (*37*–*43*). Our data show a continuous increase in the counts of MERVL-related sequences, with expression levels of MT2_Mm, the promoter of MERVL and its target genes, plateauing before those mapping to the coding sequence of the transposon, MERVL-int (fig. S3, F and G). Unlike the robust dynamics of protein-coding genes over time, we observed higher variability for TE levels between individual embryos (fig. S3H). Nonetheless, as with our findings for protein-coding genes, we observe varying expression patterns across different TEs (fig. S3I), suggesting a dynamic behavior of TEs within the span of EGA, currently of unknown functional significance.

With the increased resolution in our data, we decided to focus on DEGs between 17 and 20 hours (Fig. 3, A and E), as these could comprise early markers or regulators of subsequent embryonic transcription. We focused on factors related to epigenome regulation and identified an up-regulation of a discreet gene set pertaining to histone demethylation, encompassing the following genes: *Kdm3a, Kdm4b, Kdm4c, Kdm4d, Kdm5a, Kdm5b, Riox1,* and *Uty* (Fig. 3I). Closer inspection of their relative mRNA levels revealed that *Kdm4d* and *Uty* were lowly expressed in 2-cell stage embryos and *Riox1* was strongly up-regulated during later time points (23 and 26 hours), while the rest of the genes were continuously up-regulated as the embryo progressed through EGA (Fig. 3I). *Kdm3a*, *Kdm4b*, and *Kdm4c* encode for demethylating enzymes of histone PTMs associated with transcriptional repression (H3K9me2, H3K9me3, H3K27me3, H3K36me2, and H3K36me3), while *Kdm5a/b* is responsible for the removal of H3K4me3 (*44*). Considering that it was previously shown that ncH3K4me3 removal through Kdm5b overexpression resulted in increased BrUTP incorporation in ∼30% of the transcriptionally quiescent fully-grown oocytes (FGOs) (*22*), we hypothesized that Kdm5b overexpression from the zygote could induce a precocious remodeling of ncH3K4me3 domains, potentially alleviating its widespread repression.

**Fig. 4.**
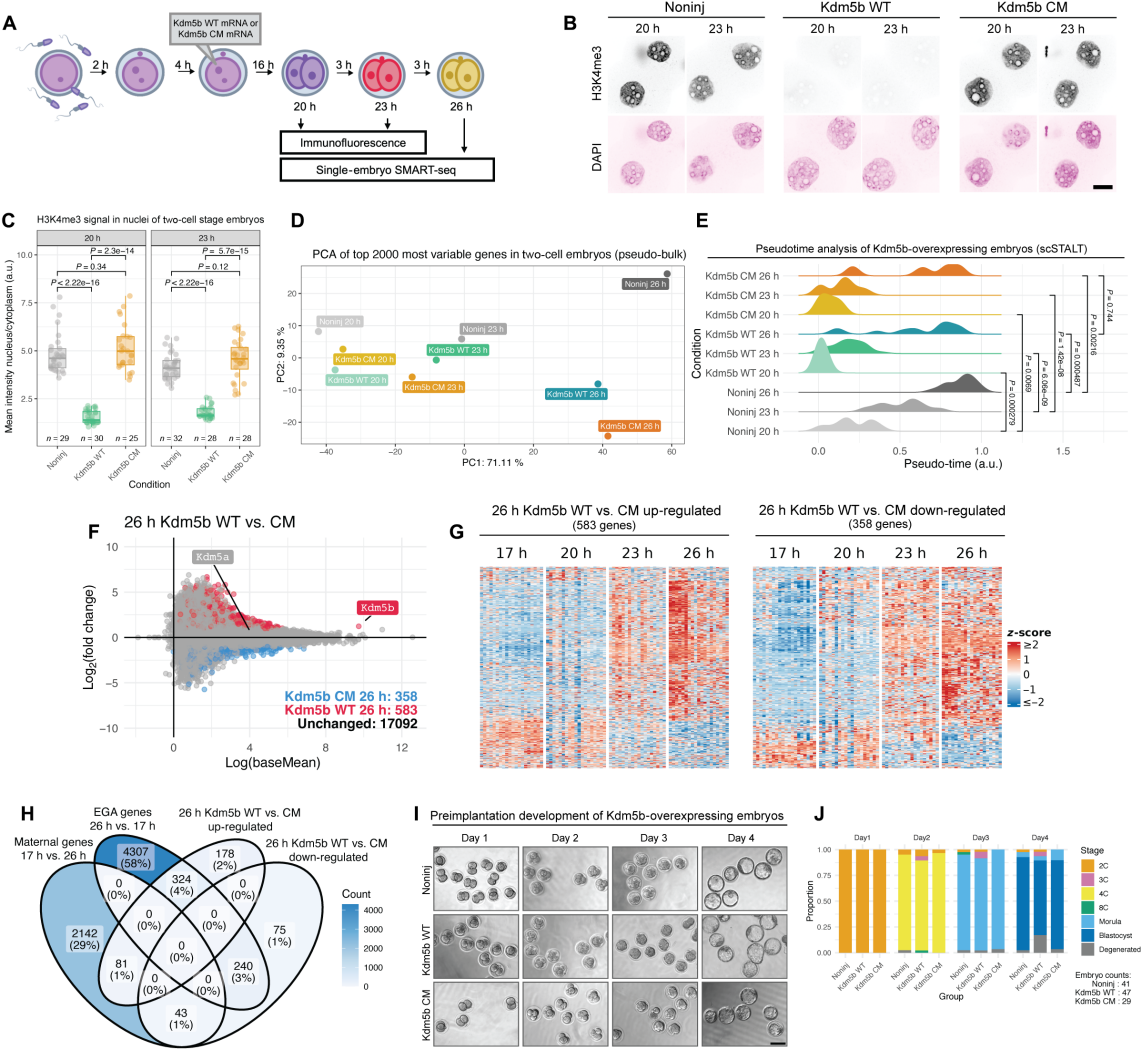
Precocious removal of H3K4me3 does not perturb EGA timing. (**A**) Schematic of the experimental setup. After IVF, zygotes were microinjected with Kdm5b WT or CM and collected at indicated time points. h, hours. (**B**) Representative immunofluorescence images of H3K4me3 at 20 and 23 hours following Kdm5b overexpression. Single z-slices are displayed. Scale bar, 20 μm. (**C**) Quantification of the data presented in (B), measured as nuclear/cytoplasmic H3K4me3 signal. Quantifications were done on z-projections of the full images. Groups were compared using Student’s *t* tests. *N* = 2 IVFs, *n* = 172 blastomeres quantified. (**D**) PCA of average counts per condition, across top 2000 most variable genes after DESeq2 counts normalization. *N* = 2 to 4 IVFs per condition, *n* = 147 total embryos analyzed. (**E**) Pseudo-time analysis of SMART-seq2 data after Kdm5b overexpression, using scSTALT. Noninj embryos were used as the reference dataset. Groups were compared using Wilcoxon rank sum tests. Distribution of the stage assignment for each condition is shown. (**F**) DESeq2 analysis of 26-hour data comparing Kdm5b WT versus CM. The number of genes enriched in each condition is indicated. (**G**) Heatmap of DEGs from (F) across the high-resolution EGA dataset visualized as *z*-scores of log(CPM + 1) counts. A total of 583 up-regulated and 358 down-regulated genes are displayed. (**H**) Venn diagram showing overlaps between DEGs from (F) and EGA (26-hour versus 17-hour up-regulated) and maternal (26-hour versus 17-hour down-regulated) genes from (Fig. 3D). (**I**) Representative images of embryos following Kdm5b microinjections at indicated time points. Scale bar, 100 μm. (**J**) Quantification of the data represented in (I). *N* = 2 IVFs, *n* = 117 embryos. Groups were compared using Fisher’s exact tests. *P* values are for the three comparisons: Noninj versus Kdm5b WT, *P* = 0.0562; Noninj versus Kdm5b CM, *P* = 0.7099; Kdm5b WT versus Kdm5b CM, *P* = 0.2568.

We examined H3K4me3 levels during EGA and found that they are gradually depleted concomitantly with EGA (Fig. 3, J and K), a pattern opposite to *Kdm5b* mRNA levels (Fig. 3I). In addition, analysis of publicly available data (*21*, *45*) confirmed that Kdm5b wild-type (WT) zygotic overexpression results in the removal of ncH3K4me3 at the early 2-cell stage (fig. S5A). Then, we assessed the impact of precocious H3K4me3 removal on the progression of EGA. To achieve this, we overexpressed Kdm5b in zygotes by microinjecting mRNA constructs coding for this enzyme (Kdm5b WT) or a catalytic mutant (CM) harboring a His^499^→Ala point mutation (*17*, *22*). As an additional control, we collected stage-matched noninjected (Noninj) embryos (Fig. 4A). Following Kdm5b overexpression and effective H3K4me3 depletion (Fig. 4, B and C, and fig. S4, A and B), we leveraged our high-resolution transcriptome mapping and performed single-embryo SMART-seq2 at 20, 23, and 26 hours after precision-IVF. PCA revealed that all groups of embryos separated along PC1 based on collection time, indicating that the embryos progressed through EGA largely uninterrupted, irrespective of the perturbation (Fig. 4D and fig. S4C).

To understand how H3K4me3 depletion affects developmental timing at the transcriptome level, we performed pseudo-time analysis using scSTALT (*46*), using Noninj embryos as the reference dataset. This revealed a developmental delay at 20 and 23 hours and, to a lesser degree, at 26 hours in microinjected samples compared to the Noninj group (Fig. 4E). The observed transcriptomic lag, rather than advancement, refuted our hypothesis that early loss of H3K4me3 may facilitate early genome reactivation. We conclude that the measured developmental delay is most likely due to the microinjection procedure rather than the premature removal of H3K4me3, as it is observed in both Kdm5b WT- and CM-injected samples. Kdm5b overexpression resulted in gene expression differences across conditions at all sampled time points (fig. S4D). We focused our analysis on 26 hours, when the developmental delay measured by scSTALT was less pronounced than at previous time points and identified 941 DEGs between Kdm5b WT and CM (Fig. 4F and table S4). Given that similar proportions of maternal and EGA genes were affected in both Kdm5b WT and CM samples, this indicated that there is no H3K4me3 depletion–specific effect on EGA timing (Fig. 4, G and H). Concordantly, we did not observe any notable differences in transcription start site (TSS) CpG density (fig. S5B). When probing gene lengths, we observed that the Kdm5b WT up-regulated genes at 26 hours were slightly longer than the down-regulated ones (fig. S5C). Promoter analysis [−1000 to +200 base pairs (bp) around the TSS] identified enrichment for transcription factors (TFs) with a strong CG signature (Sp1, Klf15, and Klf17), as well as enrichment of the Dux TF motif in the promoters of down-regulated genes, which was present in eight of the identified promoters (fig. S5, D and E). Further, probing public histone PTM data of H3K4me1, H3K9me3, H3K27ac, H3K27me3 and H3K36me3 (*47*) revealed that promoters of all tested gene sets are marked by H3K9me3 in the zygote but rapidly lose this mark one cleavage later, concomitantly with the gain of H3K27ac (fig. S6A). Collectively, we do not observe a strong, specific (epi)genomic signature associated with either up- or down-regulated gene sets, consistent with our conclusion that Kdm5b WT overexpression and precocious H3K4me3 removal do not preferentially affect specific gene expression programs during EGA. In line with this, when allowed to progress through development, both Kdm5b WT- and CM-microinjected embryos formed blastocysts at rates comparable to the Noninj embryos (Fig. 4, I and J). The same perturbation experiment, performed in FGOs, revealed no discernible separation of transcriptomes along a PCA between the experimental groups, indicating that Kdm5b overexpression and consequent H3K4me3 removal (fig. S7, A to C) did not globally alter the oocyte mRNA profiles (fig. S7D). Performing differential gene expression analysis [|log_2_(FC)| > 0.5, *P* adj. < 0.05] between Kdm5b WT-injected oocytes, and either of the two control groups further corroborated this finding, as we could detect only four DEGs besides the increased levels of Kdm5b mRNAs caused by microinjections (fig. S7, E to H). Kdm5b WT overexpression in FGOs did not increase global transcription levels, measured by the incorporation of 5-ethynyl uridine (EU), when compared to Kdm5b CM-injected or Noninj FGOs (fig. S7, I to K). In addition, H3K4me3 removal did not affect global H3K9me3 levels (fig. S7, I to L), despite the reported coregulation of H3K4me3 and H3K9me3 demethylases in FGOs (*48*). Collectively, these experiments suggest that enzymatic removal of H3K4me3 through Kdm5b overexpression in FGOs and after fertilization is not sufficient to robustly drive the reactivation of gene expression nor does it globally affect EGA timing.

## DISCUSSION

We report an easy-to-use precision-IVF protocol coupled with single-embryo RNA-seq, which is able to accurately and robustly capture the extensive transcriptome remodeling during the major wave of mouse EGA at high temporal resolution. We show that reducing the coincubation of oocytes and sperm from 4 to 2 hours increases the temporal precision of embryo staging without compromising fertilization rates or embryo quality. This protocol is a technically facile alternative to ICSI for generating high numbers of precisely staged embryos, without the requirement for specialized equipment. In addition, it reduces the experimental and biological variance caused by sperm head preparation and insertion into oocytes required by ICSI and is amenable for downstream perturbation assays, including microinjection-based delivery of RNAs or proteins. The choice of embryo generation method can affect the resulting embryonic transcriptomes. However, a comprehensive comparison of transcriptomes of 4-cell stage embryos generated by natural mating, IVF, ICSI, and parthenogenesis revealed only subtle gene expression differences when comparing IVF with natural mating. The transcriptomic differences were 10-fold higher when comparing embryos generated by natural mating to parthenotes (*49*). This indicates that IVF-derived embryos follow a healthy developmental trajectory, which is also apparent in embryos generated by precision-IVF, which develop timely into healthy blastocysts. Our data, collected from three independent biological replicates during the span of 1 year, report on the sensitivity of the precision-IVF method and its broad applicability, accurately capturing the reported continuum of gene expression changes during the 2-cell stage of development (*2*), as well as revealing previously unidentified transcriptomic dynamics during EGA.

We show that 2-cell stage embryos collected over a 9-hour time window display markedly different transcriptomes. These are reshaped as the embryo undergoes EGA, with ∼30% of all detectable genes changing in expression levels. GO analysis revealed that EGA is marked by a progressive up-regulation of genes associated with the mRNA life cycle, including those related to RNA polymerase II activity, ribosome biogenesis, and translation, as well as genes related to ubiquitin ligase activity, pointing toward a previously unappreciated step-wise progression of genome reactivation. For example, in clusters 7 to 9 (Fig. 2F), we detect genes related to ribosome biogenesis and translation, consistent with previous reports of gene classes up-regulated during EGA (*3*, *50*, *51*) (tables S1 and S2). Curiously, in cluster 9, we find factors involved in proteostasis and ubiquitylation, indicating that their up-regulation during late EGA might contribute to the degradation of maternally deposited proteins post-EGA. Instead, cluster 10 is dominated by genes related to cell cycle progression, DNA replication, and repair, which are transiently up- and down-regulated within our time course, further showcasing the sensitivity of the method in detecting rapid transcriptome changes (tables S1 and S2). We hypothesize that the diversity of temporal expression patterns detected reflects the concerted action of multiple concomitant gene regulatory mechanisms, including TF activity, changes in mRNA stability, translation rates, and epigenome remodeling. Our data are sensitive enough to detect specific pathways with distinct expression and depletion kinetics during EGA, as exemplified by processes related to tRNA metabolism, including tRNA expression and charging. These findings allow for future testing of the functional and temporal relationship between concurrent and sequential processes identified.

As EGA occurs in the context of global chromatin remodeling, we functionally probed the impact of precocious loss of maternally inherited ncH3K4me3 on the progression through EGA. Unexpectedly, after depleting H3K4me3 in FGOs across four independent biological replicates, we detected only a few significantly dysregulated genes. Although previous work reported ectopic transcriptional activity in FGOs following H3K4me3 depletion, the molecular nature of these RNAs remains unknown (*22*). Our data indicate that, once the genome has silencing has been established in FGOs, H3K4me3 removal is insufficient to reactivate transcription. This could possibly be due to the synergy between other histone PTMs, such as H3K9me3. The importance of the correct balance between H3K4me3 and H3K9me3 over ncH3K4me3 domains has previously been reported (*48*, *52*). For example, aberrant spreading of H3K9me3 over ncH3K4me3 oocyte domains leads to dampened transcriptional activation of genes and TEs during EGA, as well as chromosomal abnormalities and lower developmental rates during subsequent embryonic stages (*52*). Through immunostaining, we did not observe a global change in H3K9me3 in FGOs after Kdm5b overexpression (fig. S7I and L), which does not exclude possible subtle effects. After fertilization, early removal of H3K4me3 from chromatin had a modest but measurable impact on gene expression at different time points during EGA, strongly suggesting that H3K4me3 removal is, per se, not instructive for EGA timing, in line with previous observations (*24*, *45*). These data do not, however, exclude locus-specific roles of H3K4me3 during the investigated developmental time frame nor the possible impact of H3K4me3 dosage (globally and locally) on genome regulation after fertilization. Coupling the high temporal resolution of our dataset with pseudo-time analysis allowed us to distinguish the impact of H3K4me3 on gene expression throughout EGA from developmental delay. With thousands of genes and many TEs induced during the early stages of EGA in an unperturbed state, factoring in the influence of micromanipulation and sampling precision on developmental age is a critical consideration when interpreting the temporal and hierarchical relationships between candidate gain- or loss-of-function perturbations and transcriptomic output.

In the context of Kdm5b overexpression, we detect the biggest gene expression difference between the three experimental groups at 26 hours. Considering that all groups were able to form blastocysts at comparable rates, it is plausible that the preimplantation embryo is sufficiently plastic to buffer for untimely remodeling of certain chromatin modifications without compromising early development.

With our precision-IVF approach, we are able to resolve mouse EGA, providing a detailed dataset for the community and a means for future investigations into the underlying gene regulatory principles of EGA. Our data reveal a functional independence of EGA from H3K4me3 remodeling. Our work underscores the necessity for temporal resolution and precision when interpreting the transcriptomic readouts during EGA. Last, the high plasticity of the early embryo upon precocious loss of a histone PTM calls for further characterization of the impact of concomitant epigenetic mechanisms on transcriptional activity and developmental outcomes in vivo.

### Limitations of the study

In this study, we use the SMART-seq2 protocol to understand the mRNA changes that occur during EGA. However, the SMART-seq2 protocol cannot be used to measure absolute quantities of transcripts or the total level of transcriptional activity. This limitation can be (at least partially) circumvented by performing in vitro polyadenylation. We have coupled SMART-seq2 with our optimized precision-IVF approach, which works efficiently in FVB/NCrl strain, but further optimizations might be necessary for other mouse strains to achieve a high fertilization rate and synchronous populations of embryos. Immunostaining experiments showed that we achieved a near-complete erasure of H3K4me3 by Kdm5b overexpression. While we cannot formally exclude the possibility of residual H3K4me3 remaining on chromatin in our system, we conclude that the global removal of H3K4me3 to very low levels has minimal impact on EGA progression.

## MATERIALS AND METHODS

### Animal husbandry

Animals (*Mus musculus* FVB/NCrl strain) were housed in a stand-alone, specific pathogen–free, barrier-free facility on the European Molecular Biology Laboratory (EMBL) Rome “A. Buzzati-Traverso” campus, with separate animal and service areas and strict air-lock entry. Environmental parameters (temperature, humidity, light cycle, and ventilation) were centrally controlled and continuously monitored, and all incoming materials underwent decontamination and sterilization. Dedicated technical staff and animal caretakers followed rigorous gowning and handling procedures, while a designated veterinarian and Animal Welfare Body oversaw compliance with Legislative Decree 26/2014, EU Directive 2010/63/EU, and EMBL Policy 65. All experiments involving mice were carried out in accordance with the approved protocols and guidelines by the laboratory animal management and ethics committee of the EMBL under license no. 21-007_RM_AB and Italian Health Ministry license no. 622/2023-PR.

### In vitro fertilization

All experiments used embryos from FVB/NCrl mice. Females between 6 and 12 weeks of age were superovulated by intraperitoneal injection of 5 international unit (IU) of pregnant mare serum gonadotropin (PMSG), followed by 5 IU of hCG after 48 hours. IVF was performed according to (*53*): Males were euthanized, and the cauda epididymides and vasa deferentia were collected into human tubal fluid (HTF) medium, previously equilibrated overnight in an incubator under 20% O_2_ and 5% CO_2_ at 37°C. The spermatozoa were isolated by microdissection and incubated in a 300-μl drop of HTF containing 0.75 mM methyl-β-cyclodextrin (MBCD) under equilibrated mineral oil to allow for 1 hour for capacitation. Oocytes were collected 14 to 18 hours after hCG injection. Females were euthanized, and ampullae were collected into equilibrated potassium simplex optimized medium (KSOM). Oocytes were transferred by microdissection into a 200-μl drop of equilibrated KSOM containing 1 mM glutathione (reduced form) (GSH) under mineral oil. Insemination was performed by adding 0.2 × 10^6^ spermatozoa to each KSOM + GSH drop and incubating for 1 to 4 hours. Unless otherwise indicated, all experiments had an incubation time of 2 hours. Oocytes and zygotes (indistinguishable at this stage) were washed in equilibrated KSOM. This time point was considered 0 hours after IVF, and all subsequent time points were defined relative to this as the number of hours after IVF. Embryos were cultured in equilibrated KSOM under 20% O_2_ and 5% CO_2_ at 37°C up to the blastocyst stage.

### Isolation of FGOs

Ovaries were isolated from female mice 44 to 48 hours post–PMSG injection. Cumulus-oocyte complexes were released from the ovaries by mechanical disruption with a needle into M2 supplemented with 20 μM milrinone to prevent meiotic progression to the MII stage. Cumulus cells were removed from the FGOs by repeated washes and mouth pipetting.

### Microinjections

Microinjections were performed between 4 and 6 hours after IVF using an Eppendorf FemtoJet system with a Leica iDM8 microscope. Zygotes were washed in warm M2 medium and placed in an M2 drop under mineral oil. Kdm5b WT and CM mRNAs were microinjected into the cytoplasm of zygotes at 1.7 μg/μl, produced in-house using the mMessage mMachine T3 transcription kit from previously published plasmids (Addgene, nos. 86398 and 119767). Microinjected embryos were cultured in KSOM under 20% O_2_ and 5% CO_2_ at 37°C up to the blastocyst stage. For FGOs, the process was similar to that of zygotes, except that milrinone was added to the M2 medium during washes and microinjections to a final concentration of 20 μM. After injection, FGOs were cultured in M2 containing 20 μM milrinone and 5% fetal bovine serum (FBS) under 20% O_2_ and 5% CO_2_ at 37°C for 14 to 16 hours before collection. The concentration of mRNA used for oocyte microinjection was 1.3 to 1.7 μg/μl.

### Immunofluorescence

Embryos at the appropriate stage were washed twice in 37°C M2 medium. The zona pellucida was removed by washing embryos up to four times in drops of acidic Tyrode’s solution, followed by two washes in M2 and one wash in phosphate-buffered saline (PBS), all at 37°C. The embryos were fixed in 4% paraformaldehyde (PFA) for 20 min at 37°C and washed in PBS. The embryos were permeabilized in room temperature 0.5% Triton X-100 in PBS and washed thrice in room temperature PBS-Tween 0.5% (PBSt) for 5 to 10 min. Unspecific epitopes were blocked using 3% bovine serum albumin (BSA) in PBSt for 1 to 2 hours at 37°C. The embryos were incubated overnight with primary antibody diluted 1:200 in 3% BSA in PBSt at 4°C. The following day, the embryos were washed thrice in room temperature PBSt for 5 to 10 min. A secondary blocking step was performed in 3% BSA in PBSt for 30 min at room temperature. The secondary antibody was used at a 1:500 dilution in 3% BSA in PBSt. The embryos were incubated in secondary antibodies at room temperature for 2 to 4 hours and protected from light. Last, the embryos were washed three times in PBSt for 5 to 10 min and mounted on glass slides using 10 μl of VECTASHIELD + 4′,6-diamidino-2-phenylindole (DAPI). The images were acquired using a 60× objective on a Nikon Crest V3 spinning disk microscope. Z-stacks were acquired in 1-μm slices.

### EU incorporation in FGOs

At ∼16 hours, FGOs were incubated for 2 hours in 100-μl drops of M2 supplemented with 20 μM milrinone and 500 μM EU, ensuring even distribution of oocytes between drops. This step was performed under equilibrated mineral oil in an incubator under 20% O_2_ and 5% CO_2_ at 37°C. The EU used was from the Click-iT Nascent RNA Capture Kit. FGOs were washed three times in M2 supplemented with 20 μM milrinone. The zona pellucida was removed using acidic Tyrode’s solution, and the oocytes were fixed in 4% PFA for 20 min at 37°C, washed in PBS, and permeabilized in 0.5% Triton X-100 for 20 min at room temperature. Subsequently, three PBSt washes of 10 min were performed at room temperature. For each sample, 50 μl of Click-IT reaction was prepared according to the manufacturer’s instructions using the reagents from the Click-IT EdU Cell Proliferation Kit. The Click-IT reaction was set up as follows: 42.8 μl of Click-IT reaction buffer, 2 μl of CuSO_4_, 0.2 μl of Alexa Fluor 488 azide, and 5 μl of 1× Click-IT reaction buffer additive. The embryos were incubated for 45 min in 50 μl of Click-IT reaction at room temperature in the dark. All subsequent steps were performed in the dark. The samples were washed once in the rinse buffer from the kit and twice in PBSt. Each wash was 10 min at room temperature. The samples were blocked in 3% BSA in PBSt for 1 hour at 37°C and incubated with primary antibodies diluted in 3% BSA in PBSt overnight at 4°C. The experiment was completed using the regular immunofluorescence protocol described above. The nuclear configuration (SN or NSN) of each oocyte was classified upon imaging.

### Image quantifications

Image analysis of the immunofluorescence data was performed using FIJI. Depending on the experiment, different image analysis approaches were selected, as detailed in the figure legends. For single z-slice analysis, the z-slice containing the widest part of the nucleus of each blastomere was chosen for quantification. The nucleus was selected by thresholding the DAPI signal, ensuring that it was captured as a single continuous region. The signal from the antibody target was measured relative to the intensity of DAPI staining. Mean intensity scores were used. For full z-stack analyses, a z-projection using SD was generated for each channel, and the mean signal in the nucleus and cytoplasm was measured. In these cases, a nuclear-to-cytoplasmic ratio for each channel was calculated. Polar bodies were discarded from the analyses.

### Single-embryo/oocyte SMART-seq2

At the appropriate stage, embryos or oocytes were washed in M2 prewarmed to 37°C. The embryos or oocytes were collected individually using a mouth pipette and lysed on ice in 5 μl in a lysis buffer containing 0.5% Triton X-100, 2.5 mM deoxynucleotide triphosphates (dNTPs), 2.5 μM anchored oligo(dT), and SUPERase-In ribonuclease (RNase) inhibitor (0.5 U/μl). Samples were stored at −20°C until library preparation. For SMART-seq2 + PolyA, embryos were collected individually using a mouth pipette and lysed in 10 μl in 1× TCL buffer containing 1% β-mercaptoethanol. Samples were stored at −80°C until library preparation. Briefly, RNA was purified using a 2.2× ratio of RNAClean XP beads. Beads were washed three times with 80% ethanol, and then RNA was eluted in 10 μl of RNase-free water. From each sample, 5 μl of RNA underwent in vitro polyadenylation, and 5 μl were subjected to the same processing, but omitting the poly(A) polymerase. RNA was mixed with 1 μl of 10× *Escherichia coli* poly(A) polymerase reaction buffer, 1 μl of 10 mM adenosine 5′-triphosphate (ATP), 0.25 μl of SUPERase RNase inhibitor, 2.5 μl of RNase-free water, and 0.25 μl of *E. coli* poly(A) polymerase. Samples were incubated at 37°C for 30 min and then kept at 4°C. Then RNA was bead purified again as described above with a 2.2× ratio of RNAClean XP beads and eluted in 10 μl of RNase-free water containing 2.5 mM dNTPs and 2.5 μM anchored oligo(dT). For reverse transcription and subsequent library preparation steps, the protocol for regular SMART-seq2 was followed, as described below.

SMART-seq2 libraries were prepared by the EMBL Genecore Facility, as previously described (*25*, *26*). First, oligo-dT was annealed to the mRNA by incubating 3 min at 72°C. Subsequently, for the reverse transcription, the following reagents were added to each sample: 2 μl of SSRT IV 5× buffer, 2 μl of 5 M betaine, 0.50 μl of 100 mM dithiothreitol, 0.1 μl of 1 M MgCl_2_, 0.25 μl of RNase inhibitor (40 U/μl), 0.25 μl of SuperScript IV reverse transcriptase (200 U/μl), 0.1 μl of 100 μM template-switching oligo, and 1.15 μl of RNase-free water. Reverse transcription was performed by incubating the samples for 15 min at 52°C, followed by 10 min at 80°C and a 10°C hold. Reverse transcription was immediately followed by 20 cycles of whole-transcriptome amplification: 12.5 μl of KAPA HiFi HotStart ReadyMix 2× and 2.50 μl of RNase-free water were added to each sample and amplified under the following polymerase chain reaction (PCR) cycling conditions: denaturation for 3 min at 98°C; 20 cycles of 20-s denaturation at 98°C, 15-s annealing at 67°C, and 6-min elongation at 72°C; and a 5-min final elongation at 72°C, followed by a 10°C hold. Samples were cleaned up using a 0.6× ratio of SPRIselect beads. Ethanol washes were omitted, and samples were directly eluted in 13 μl of RNase-free water.

Homemade Tn5 produced by EMBL Pepcore was complexed with mosaic end adapters (Tn5ME-A/rev and Tn5ME-B/rev), as described previously (*26*), and was used for tagmentation of the samples. cDNA samples were diluted to a range of 0.1 to 0.2 ng/μl. For tagmentation, the following components were mixed: 1.25 μl of diluted sample, 2.5 μl of freshly prepared tagmentation mix [20 mM tris-HCl (pH 7.5), 20 mM MgCl_2_, and 50% *N*,*N*′-dimethylformamide (DMF)], and 1:300 homemade Tn5 with mosaic end adapters in a final volume of 5 μl. Tagmentation was performed at 55°C for 3 min. Tagmentation was stopped by the addition of 1.25 μl of 0.2% SDS and followed by a final PCR amplification. A total of 6.75 μl of KAPA HiFi HotStart ReadyMix 2×, 0.75 μl of 100% dimethyl sulfoxide, 2.5 μl of 2.5 mM i5 adapter index primer, and 2.5 μl of 2.5 mM i7 adapter index primer were added to each sample and amplified under following conditions: 3 min at 72°C, 30 s at 95°C, and 12 cycles of 20 s at 98°C, 15 s at 58°C and 30 s at 72°C, followed by a final elongation of 3 min at 72°C and a 10°C hold. Samples were pooled, and the final pool was purified using 0.8× SPRIselect bead purification. The size distribution of the pool was checked with an Agilent 2100 Bioanalyzer using the Agilent High Sensitivity DNA Kit and quantified using the Qubit double-stranded DNA (dsDNA) high-sensitivity assay. All sequencing of 2-cell datasets was paired-end and performed on an Illumina NextSeq 500. The samples were sequenced paired-end with 40 bp for each read. For FGOs, the samples were sequenced on Illumina NextSeq 2000 using 50-bp paired-end sequencing. The samples were sequenced to an average depth of ∼5 million reads per sample.

### SMART-seq2 data analysis for genes

Fastq files were mapped to the GRCm38/mm10 genome containing only major chromosomes (chr1 to chr19, X, Y, and MT) using STAR. For SMART-seq2 data generated in this study, paired-end reads were mapped using alignreads mode with the following parameters: --outFilterMultimapNmax 20 --outFilterMultimapScoreRange 0 --outFilterMismatchNoverLmax 0.05 --sjdbScore 2. Gene counts were quantified using featureCounts from Subread with parameters -p and –countReadPairs.

*z*-scores of log(CPM + 1) values were calculated using the EdgeR package values and used for heatmap visualization with the ComplexHeatmap package. TPMs were calculated manually on the basis of raw counts.

DEGs were obtained using DESeq2. Because of the time-resolved nature of our data, DESeq2 was run using the likelihood ratio test, where the full model contained the condition parameter, which was removed in the reduced model. For the EGA time course, each time point was considered a condition. For the Kdm5b overexpression dataset, the condition consisted of a concatenation of the time point and the experimental condition. For FGOs, a Wald test was used with the experimental condition as the design parameter. ClusterProfiler was used downstream to perform GO analysis. Hierarchical clustering was computed with DESeq2 normalized counts using Euclidean distances.

PCAs were plotted using variance-stabilized (VST) counts from the DESeq2 analysis, which were corrected for batch effects using removeBatchEffect from the limma package. The top 2000 most variable genes were considered for the PCAs. For the Kdm5b overexpression dataset, DESeq2 normalized counts were averaged per condition before visualizing the PCA.

Strictly embryonic genes were defined by intersecting published EGA genes (*54*), and genes significantly up-regulated [log_2_(FC) > 1 and *P* adj. < 0.05] from 18 to 28 hours (*2*). Strictly maternal genes were defined as the ones significantly down-regulated [log_2_(FC) < −1 and *P* adj. < 0.05] from 18 to 28 hours (*2*). These gene lists were generated as in (*28*).

For fuzzy *c*-means clustering of genes over time, we used the mfuzz package. We combined DEGs from all pairwise comparisons and removed lowly expressed DEGs with a TPM sum of medians of <5 to obtain well-defined clusters. Clustering was done on DESeq2 normalized counts using parameters *c* = 12 and *m* = 1.8. The membership score is calculated for each gene across clusters, and the gene is then assigned to the cluster with the highest membership score. The membership score ranges between 0 (poor membership) and 1 (perfect membership), with the membership score sum for each gene across all clusters being 1. As genes vary in their expression dynamics, we opted for fuzzy clustering rather than hard (*k*-means) clustering when examining the many genes that change in our time course.

scSTALT was performed according to (*46*). The kernel was run on Noninj data using parameters nGenes = 5000, nKnots = 4, nPoints = 100, and fdr = 0.05. Kdm5b embryos were then stage assigned onto the reference data, and the distribution of the stage assignment is visualized.

To identify TSS locations, we used the ENSEMBL mm10 biomart annotation. TSS was defined as the first annotated base pair of each gene on the + strand and as the last annotated base pair on the − strand. Gene lengths were calculated on the basis of the same annotation reference as the difference between the end and the start coordinates of each gene. One annotation (TSS or gene length) per gene was used.

TF motif enrichment was performed using HOMER. A promoter window of −1000 and +200 bp relative to the TSS of each gene was defined using BEDTools slop in a strand-sensitive manner. HOMER findMotifsGenome.pl was run with parameters mm10 and -size given.

### SMART-seq2 data analysis of TEs

To estimate TE expression, mapping parameters were adjusted: STAR was run using parameters: --outFilterMultimapNmax 5000 --outSAMmultNmax 1 --outFilterMismatchNmax 3 --outMultimapperOrder Random --winAnchorMultimapNmax 5000 --alignEndsType EndToEnd --alignIntronMax 1 --alignMatesGapMax 350 --seedSearchStartLmax 30 --alignTranscriptsPerReadNmax 30000 --alignWindowsPerReadNmax 30000 --alignTranscriptsPerWindowNmax 300 --seedPerReadNmax 3000 --seedPerWindowNmax 300 --seedNoneLociPerWindow 1000. Location of TEs was determined using the GRCm38/mm10 RepeatMasker annotation after removal of low complexity and simple repeats. Mapped reads were filtered such that only instances with both paired reads fully overlapping with the TE were considered for downstream analysis. Reads overlapping with gene bodies were also discarded to ensure that the read stemmed from the TE and not from the expression of an overlapping gene. Read quantification was performed using featureCounts and options -p -B -s 0 --fracOverlap 1 -M to summarize across repeat names. For downstream analysis, all reads belonging to each repeat name were counted.

To identify differentially expressed TEs, we used a log_2_(FC) threshold of ±0.5 and a *P* adj. value threshold of <0.01 using DESeq2 and a likelihood ratio test. Expression levels were plotted using DESeq2 normalized counts.

Fuzzy *c*-means clustering was performed as described above, using the mfuzz package with parameters *c* = 9 and *m* = 1.8. No expression level filter was used for TEs.

### Analysis of publicly available data

Publicly available SMART-seq data from (*27*) was downloaded from GSE45719. SMART-seq libraries were mapped using STAR and parameters --outFilterMultimapNmax 20 --outFilterMultimapScoreRange 0 --outFilterMismatchNoverLmax 0.05 --sjdbScore 2, using single reads as inputs. Reads were counted using featureCounts and analyzed together with the featureCounts data from the EGA time course SMART-seq2 data. Genes with a total row sum of less than 200 were filtered out. PCA of DESeq2 normalized counts was plotted over the top 2000 most variable genes.

H3K4me3 CUT&Tag data in early 2-cell stage embryos upon Kdm5b WT and Kdm5b CM overexpression was downloaded from CRA015496 (*52*) and processed with the nf-core cutandrun v3.2.2. pipeline with parameters: dedup_target_reads: true\normalisation_mode: Spikein\normalisation_binsize: 10\ spikein_genome: BDGP6 \dump_scale_factors: true\extend_fragments: true\save_spikein_aligned: true\minimum_alignment_q_score: 30. Mapping was performed using Bowtie2 and mapping only to major chromosomes (chr1 to chr19, X, Y, and MT). For blacklisted regions, we used the standard mm10 blacklist regions and removed from the blacklist the parts of the regions that contained DEGs from our EGA time course (across all comparisons). CUT&Tag coverage was visualized using deepTools across specified regions using *D. melanogaster* spike-in normalized reads.

To define ncH3K4me3 regions, publicly available H3K4me3 micro–chromatin immunoprecipitation sequencing data were downloaded from GSE72784 (*21*). The raw fastq files were trimmed with Trimmomatic (−phred33 LEADING:3 TRAILING:3 SLIDINGWINDOW:4:15 MINLEN:21) and mapped the trimmed data with Bowtie2 and parameters -X 1000 --fr --no-mixed --no-discordant --dovetail --very-sensitive. SAMtools is used to discard unmapped and low-quality mapped reads with -F 4 -q 10. Peak calling was performed using MACS2 with parameters -B -m 5 50 -p 1e-5 --broad --keep-dup all. Peak calling was performed using matching replicate input samples as controls. Peaks within 5 kB were merged within each replicate using BEDTools intersect, and the replicates were then intersected to define common peaks. ncH3K4me3 domains were defined as common peaks over 5 kB.

The location of CpG islands and observed/expected CpG values was downloaded from UCSC table browser using the mm10 “CpG Islands” track. The output bed file was converted to a bedGraph format, retaining CpG island locations and observed/expected CpG values. The .bedGraph file was then converted to a .bigwig file using the bedGraphToBigWig tool for visualization. Visualization of CpG islands and observed/expected ratios over TSSs was performed using deepTools.

To probe the signal of different histone PTMs over TSSs of DEGs, processed pseudo-bulk .bigwig files were downloaded from GSE235109 (*47*). These data had already been mapped to the mm10 genome, and we visualized TACIT coverage across TSSs of specified gene sets using deepTools. All additional details regarding reagents, experimental models, data sharing, and software and algorithms used in this study are outlined in Table 1.

**Table 1.** Resource and data access table associated with the manuscript.

Reagent or resource	Source	Identifier
Antibodies		
Anti-H3K4me3 (rabbit)	Diagenode	Catalog no. c15410003
Anti-H3K4me3 (mouse)	Diagenode	Catalog no. C15200152
Anti-H3K9me3	Abcam	Catalog no. ab8898
Goat anti-rabbit immunoglobulin G (IgG) (H + L) cross-adsorbed secondary antibody, Alexa Fluor 555	Thermo Fisher Scientific	Catalog no. A-21428
Goat anti-rabbit IgG (H + L) cross-adsorbed secondary antibody, Alexa Fluor 647	Thermo Fisher Scientific	Catalog no. A-21245
Donkey anti-mouse IgG (H + L) cross-adsorbed secondary antibody, Alexa Fluor 555	Thermo Fisher Scientific	Catalog no. A-31570
Chemicals/reagents		
Recombinant RNase Inhibitor (40 U/μl)	Clontech Takara	Catalog no. 2313A
PMSG	ProSpec Bio	Catalog no. HOR-272 (5000 IU)
hCG	Merck	Catalog no. CG5-1VL
EmbryoMaxKSOM mouse embryo medium	Merck	Catalog no. MR-106-D
EmbryoMaxHTF	Merck	Catalog no. MR-070-D
MBCD	Merck	Catalog no. C4555-1G
GSH	Merck	Catalog no. G6013-5G
M2 medium	Merck	Catalog no. M7167-50ML
Triton X-100	Merck	Catalog no. T9284-100ML
Pierce 16% formaldehyde (w/v), methanol-free (16% PFA)	Thermo Fisher Scientific	Catalog no. 28908
Dulbecco’s PBS, no calcium, no magnesium (PBS, 1×)	Thermo Fisher Scientific	Catalog no. 14190169
Ambion nuclease-free water (not diethyl pyrocarbonate–treated)	Thermo Fisher Scientific	Catalog no. AM9932
Tween 20	Merck	Catalog no. P9416-100ML
BSA	Merck	Catalog no. A7906
VECTASHIELD antifade mounting medium with DAPI	Vector Laboratories	Catalog no. H-1200-10
Milrinone	Thermo Fisher Scientific	Catalog no. J62659.MA
FBS	Thermo Fisher Scientific	Catalog no. 10270106
Set of 2′-deoxyadenosine 5′-triphosphate, 2′-deoxycytidine 5′-triphosphate, 2′-deoxyguanosine 5′-triphosphate, and 3′-deoxythymidine 5′-triphosphate	Promega	Catalog no. U1420
SUPERase·In RNase inhibitor (20 U/μl)	Thermo Fisher Scientific	Catalog no. AM2696
EmbryoMax acidic Tyrode′s solution	Merck	Catalog no. MR-004-D
SPRIselect beads	Beckman Coulter	Catalog no. B23319
RNAClean XP beads	Beckman Coulter	Catalog no. A63987
Betaine	Merck	Catalog no. B0300-1VL
MgCl_2_	Ambion	Catalog no. AM9530G
DMF	Merck	Catalog no. 227056
Buffer TCL	QIAGEN	Catalog no. 1070498
Tn5	EMBL Pepcore	NA
2-mercaptoethanol	Merck	Catalog no. M6250
Critical commercial assays		
mMESSAGE mMACHINE T3 transcription kit	Thermo Fisher Scientific	Catalog No. AM1348
SuperScript IV reverse transcriptase	Thermo Fisher Scientific	Catalog No. 18091050
Roche Diagnostics KAPA HiFi HotStart ReadyMix	Thermo Fisher Scientific	Catalog No. 50-196-5299
*E. coli* poly(A) polymerase	New England Biolabs	Catalog no. M0276L
Qubit dsDNA quantitation, high sensitivity	Thermo Fisher Scientific	Catalog no. Q32854
High-sensitivity DNA bioanalyzer kit	Agilent	Catalog no. 5067-4626
i5- and i7-indexed adapters	IDT	Custom for EMBL Genecore
Click-iT nascent RNA capture kit, for gene expression analysis	Thermo Fisher Scientific	Catalog no. C10365
Click-iT EdU cell proliferation kit for imaging, Alexa Fluor 488 dye	Thermo Fisher Scientific	Catalog no. C10337
Deposited data		
E-MTAB-15452	This study	www.ebi.ac.uk/biostudies/arrayexpress/studies/E-MTAB-15452?key=5b45bf1b-d2e3-407b-933c-e688f09bd127
E-MTAB-15453	This study	www.ebi.ac.uk/biostudies/arrayexpress/studies/E-MTAB-15453?key=455d8841-1ade-420f-b06e-674eb88aa30b
E-MTAB-15454	This study	www.ebi.ac.uk/biostudies/arrayexpress/studies/E-MTAB-15454?key=0b75e0f9-69d0-415b-99cb-b6bbfa762d29
E-MTAB-16935	This study	www.ebi.ac.uk/biostudies/arrayexpress/studies/E-MTAB-16935?key=3abb8130-440b-4bdc-a775-1c7d8b0486fd
Experimental models		
FVB/NCrl	EMBL Rome Laboratory Animal Resources	EMBL IACUC code 21-007_RM_AB
Oligonucleotides		
Anchored oligo(dT)	IDT	5′-AAGCAGTGGTATCAACGCAGAGTACTTTTTTTTTTTTTTTTTTTTTTTTTTTTTTVN-3′
Template switching oligo	Exicon	5′-AAGCAGTGGTATCAACGCAGAGTACATrGrG+G-3′
Tn5ME-A	IDT	5′-TCGTCGGCAGCGTCAGATGTGTATAAGAGACAG-3′
Tn5ME-B	IDT	5′-GTCTCGTGGGCTCGGAGATGTGTATAAGAGACAG-3′
Tn5MErev	IDT	5′-[phos]CTGTCTCTTATACACATCT-3′
Recombinant DNA		
RN3P-KDM5B-FLAG	Addgene	Plasmid no. 86398
pRN3P_Kdm5b_MUT	Addgene	Plasmid no. 119767
Software and algorithms		
FIJI	Schneider *et al.* (*55*)	
FASTQC v.0.11.9	bioinformatics.babraha.ac.uk/projects/fastqc/	
MultiQC v.1.12	Ewels *et al.* (*56*)	
STAR v.2.7.9a	Dobin *et al.* (*57*)	
SAMtools v.1.14	Danecek *et al.* (*58*)	
deepTools v.3.5.5	Ramirez *et al.* (*59*)	
Subread v.2.0.3	Liao *et al.* (*60*)	
R v4.2.2 and v4.4.1	R Core Team (2022)	www.R-project.org/
Tidyverse v2.0.0	https://cran.r-project.org/	https://doi.org/10.21105/joss.01686
AnnotationDbi v1.60.2	https://bioconductor.org/	DOI: 10.18129/B9.bioc.AnnotationDbi
org.Mm.eg.db v.3.16.0	https://bioconductor.org/	DOI: 10.18129/B9.bioc.org.Mm.eg.db
DESeq2 v1.44.0	Love *et al.* (*33*)	
ComplexHeatmap v.2.20.0	Gu *et al.* (*61*)	
pheatmap v.1.0.12	https://cran.r-project.org/	DOI: 10.32614/CRAN.package.pheatmap
ggsignif v0.6.4	https://cran.r-project.org/	DOI: 10.32614/CRAN.package.ggsignif
ClusterProfiler v.4.12.0	Wu *et al.* (*62*)	
edgeR v4.2.0	Robinson *et al.* (*63*)	
Mfuzz v.2.64.0	Futschik and Carlisle (*64*)	
limma v.3.60.3	Ritchie *et al.* (*65*)	
Seurat v4.3.0	Hao *et al.* (*66*)	
tradeSeq v.1.12.0	Van den Berge *et al.* (*67*)	
Harmony v.0.1.1	Korsunsky *et al.* (*68*)	
Scater v.1.26.1	McCarthy *et al.* (*69*)	
Slingshot v.2.6.0	Street *et al.* (*70*)	
scSTALT v.0.1.0	Luo *et al.* (*46*)	
MLmetrics v.1.1.1	https://cran.r-project.org/	www.rdocumentation.org/packages/MLmetrics/versions/1.1.1
ggVennDiagram v.1.5.3	https://cran.r-project.org/	https://gaospecial.github.io/ggVennDiagram/
BEDTools v.2.30.0	Quinlan and Hall (*71*)	
Trimmomatic v.0.39	Bolger *et al.* (*72*)	
Bowtie2 v.2.4.4	Langmead and Salzberg (*73*)	
nf-core cutandrun v.3.2.2	Ewels *et al.* (*74*)	https://nf-co.re/cutandrun/3.2.2
HOMER v.5.1	Heinz *et al.* (*75*)	http://homer.ucsd.edu/homer/
MACS2 v. 2.2.7.1	Zhang *et al.* (*76*)	
