# Supplementary material for: High-resolution mapping of embryonic genome activation unveils a decoupling of transcription activation from precocious H3K4me3 removal
Source: Sci Adv. 2026 Aug 7;12(32):eaec9545. doi: 10.1126/sciadv.aec9545 (PMC13450217; doi:10.1126/sciadv.aec9545)
Supplement: Supplementary file 1 [file sciadv.aec9545_sm.pdf]

Science Advances
AAAS

Supplementary Materials for

High-resolution mapping of embryonic genome activation unveils a decoupling of transcription activation from precocious H3K4me3 removal

Jasmina Al-Mousawi *et al.*

Corresponding author: Ana Boskovic, ana.boskovic@embl.it

Sci. Adv. **12**, eaec9545 (2026)
DOI: 10.1126/sciadv.aec9545

The PDF file includes:

Figs. S1 to S7
Legends for tables S1 to S5

Other Supplementary Material for this manuscript includes the following:

Tables S1 to S5

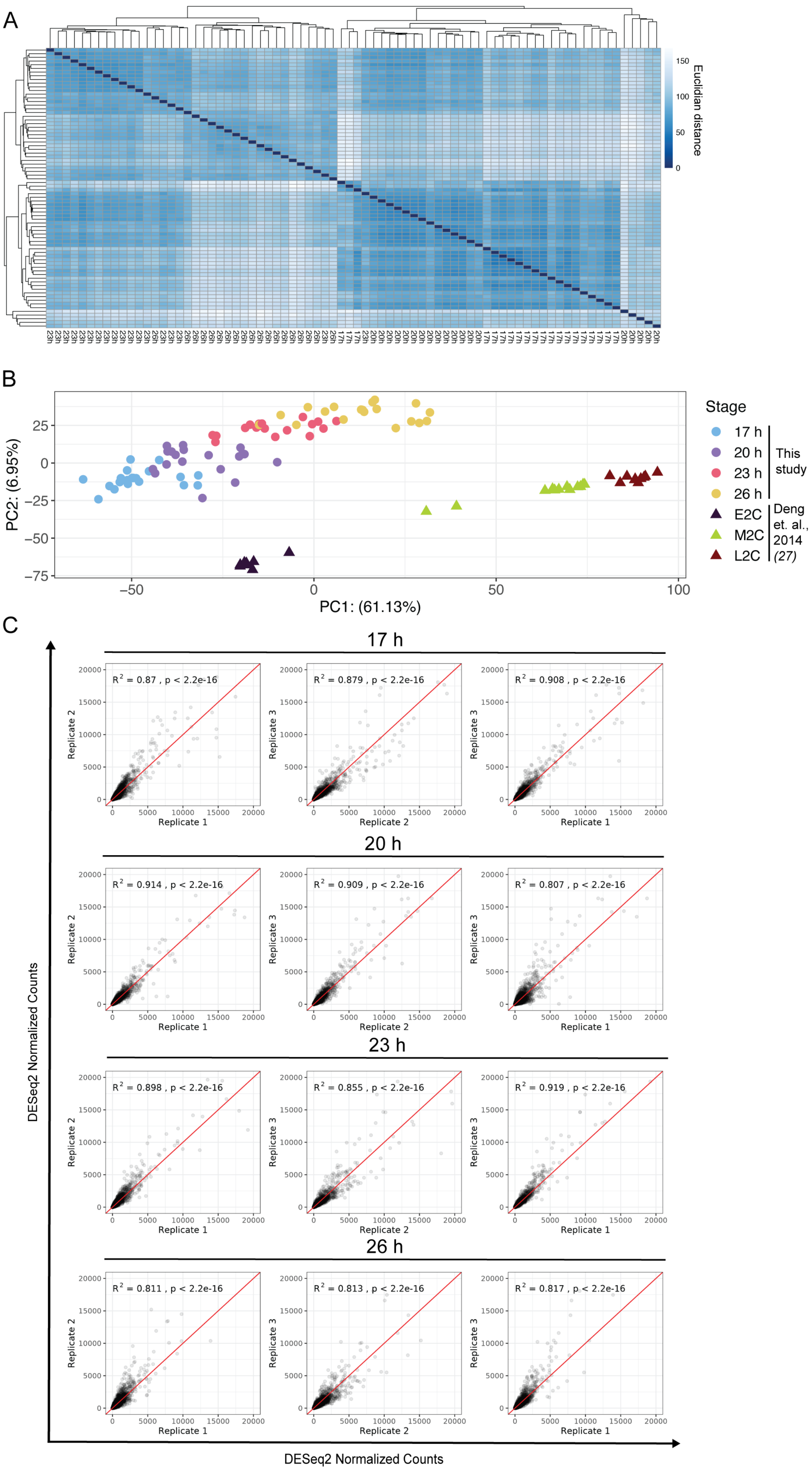
A
Euclidian distance
150
100
50
0
B
Stage
17 h
20 h
23 h
26 h
This study
E2C
M2C
L2C
Deng et. al., 2014 (27)
PC2: (6.95%)
PC1: (61.13%)
C
17 h
20 h
23 h
26 h
R² = 0.87 , p < 2.2e-16
R² = 0.879 , p < 2.2e-16
R² = 0.908 , p < 2.2e-16
R² = 0.914 , p < 2.2e-16
R² = 0.909 , p < 2.2e-16
R² = 0.807 , p < 2.2e-16
R² = 0.898 , p < 2.2e-16
R² = 0.855 , p < 2.2e-16
R² = 0.919 , p < 2.2e-16
R² = 0.811 , p < 2.2e-16
R² = 0.813 , p < 2.2e-16
R² = 0.817 , p < 2.2e-16
Replicate 1
Replicate 2
Replicate 3
DESeq2 Normalized Counts

Fig. S1. High-Resolution Mapping of Mouse Embryonic Genome Activation. A. Hierarchical clustering of individual embryos using DESeq2 normalized counts. **B.** Principal Component Analysis integrated with 2-cell data from(27). The top 2000 most variable genes are shown after DESeq2 count normalization and variance stabilizing transformation. Legend annotations: E2C: early-2-cell; M2C: mid-2-cell and L2C: late-2-cell. **C.** Comparison of mean gene expression across biological replicates, for each analyzed time point. The red lines display x=y, and Pearson's correlations have been calculated. DESeq2 normalized counts were used, and genes with mean expression > 20000 have been filtered out prior to analysis.

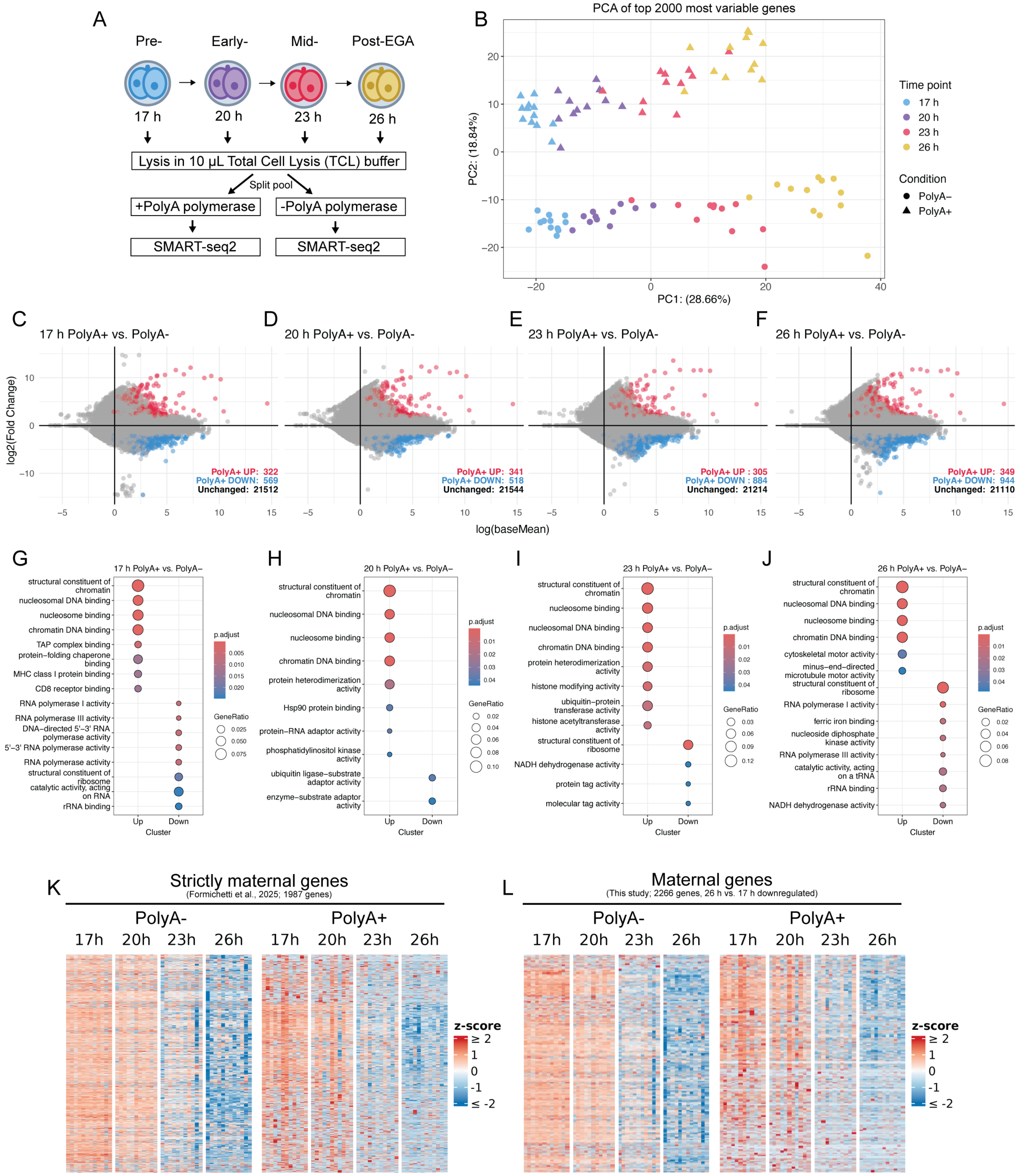

A
Pre-
Early-
Mid-
Post-EGA
17 h
20 h
23 h
26 h
Lysis in 10 µL Total Cell Lysis (TCL) buffer
Split pool
+PolyA polymerase
-PolyA polymerase
SMART-seq2
SMART-seq2
B
PCA of top 2000 most variable genes
PC2: (18.84%)
PC1: (28.66%)
Time point
17 h
20 h
23 h
26 h
Condition
PolyA−
PolyA+
C
17 h PolyA+ vs. PolyA-
PolyA+ UP: 322
PolyA+ DOWN: 569
Unchanged: 21512
D
20 h PolyA+ vs. PolyA-
PolyA+ UP: 341
PolyA+ DOWN: 518
Unchanged: 21544
E
23 h PolyA+ vs. PolyA-
PolyA+ UP : 305
PolyA+ DOWN : 884
Unchanged: 21214
F
26 h PolyA+ vs. PolyA-
PolyA+ UP: 349
PolyA+ DOWN: 944
Unchanged: 21110
log2(Fold Change)
log(baseMean)
G
17 h PolyA+ vs. PolyA−
structural constituent of chromatin
nucleosomal DNA binding
nucleosome binding
chromatin DNA binding
TAP complex binding
protein−folding chaperone binding
MHC class I protein binding
CD8 receptor binding
RNA polymerase I activity
RNA polymerase III activity
DNA−directed 5'−3' RNA polymerase activity
5'−3' RNA polymerase activity
RNA polymerase activity
structural constituent of ribosome
catalytic activity, acting on RNA
rRNA binding
H
20 h PolyA+ vs. PolyA−
structural constituent of chromatin
nucleosomal DNA binding
nucleosome binding
chromatin DNA binding
protein heterodimerization activity
Hsp90 protein binding
protein−RNA adaptor activity
phosphatidylinositol kinase activity
ubiquitin ligase−substrate adaptor activity
enzyme−substrate adaptor activity
I
23 h PolyA+ vs. PolyA−
structural constituent of chromatin
nucleosome binding
nucleosomal DNA binding
chromatin DNA binding
protein heterodimerization activity
histone modifying activity
ubiquitin−protein transferase activity
histone acetyltransferase activity
structural constituent of ribosome
NADH dehydrogenase activity
protein tag activity
molecular tag activity
J
26 h PolyA+ vs. PolyA−
structural constituent of chromatin
nucleosomal DNA binding
nucleosome binding
chromatin DNA binding
cytoskeletal motor activity
minus−end−directed microtubule motor activity
structural constituent of ribosome
RNA polymerase I activity
ferric iron binding
nucleoside diphosphate kinase activity
RNA polymerase III activity
catalytic activity, acting on a tRNA
rRNA binding
NADH dehydrogenase activity
p.adjust
GeneRatio
Up
Down
Cluster
K
Strictly maternal genes
(Formichetti et al., 2025; 1987 genes)
PolyA-
PolyA+
17h
20h
23h
26h
z-score
≥ 2
1
0
-1
≤ -2
L
Maternal genes
(This study; 2266 genes, 26 h vs. 17 h downregulated)
PolyA-
PolyA+
17h
20h
23h
26h
z-score
≥ 2
1
0
-1
≤ -2

Fig. S2. Impact of Experimental Polyadenylation on Transcriptome Detection during Mouse Embryonic Genome Activation. A. Schematic of the experimental procedure. Each lysed embryo sample was split in half and in vitro polyadenylation was performed on half of the sample. SMART-seq2 libraries were built of PolyA+ and PolyA-samples in parallel. **B.** Principal Component Analysis of single embryos sequenced using SMART-seq2, with or without experimental polyadenylation. The panel shows the top 2000 most variable genes after DESeq2 count normalization and variance stabilizing transformation. N = 2 IVFs, n = 46 embryos, each represented twice (PolyA+ and PolyA-). **C-F.** Gene expression differences comparing PolyA- and PolyA+ samples within each timepoint, determined by DESeq2 analysis using $|Log2(FC)| > 2$ and padj. < 0.05 for all comparisons. The baseMean value is the mean expression level of each gene calculated based on DESeq2 normalized counts across all samples in the dataset. Legend labels indicate the number of genes enriched in each condition. **G-J.** Gene Ontology analysis of the differentially expressed genes from panels E-H using ClusterProfiler. The Molecular Function category is displayed. Top 8 most significant terms per condition are shown. K-L. Heatmaps of strictly maternal genes curated in(28) (K, 1987 genes displayed) and maternal genes identified in this study (L, 2266 genes displayed, 26 h vs. 17 h downregulated from (Fig. 3D)), visualizing z-scores of log(CPM+1) counts.

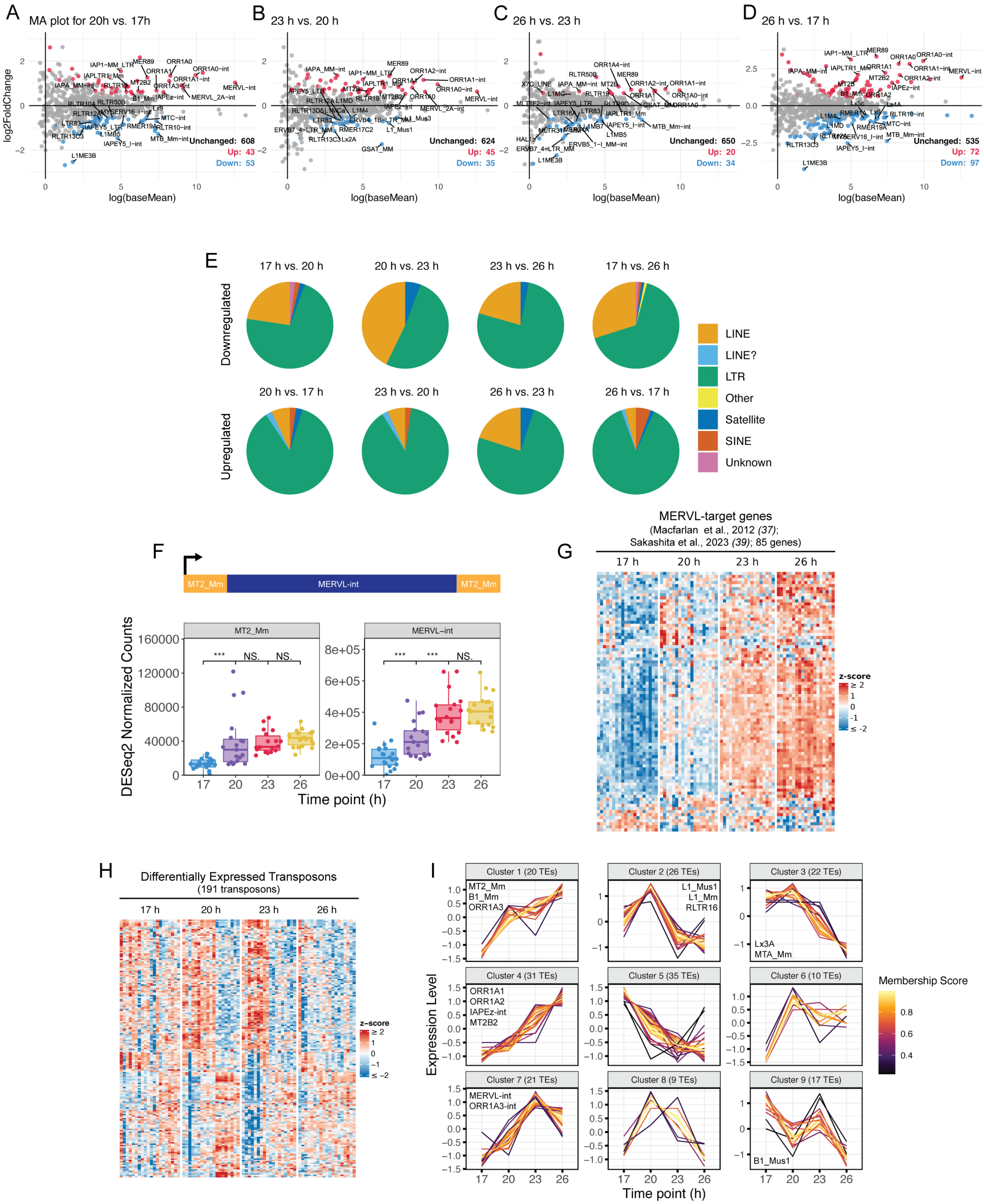

A
MA plot for 20h vs. 17h
B
23 h vs. 20 h
C
26 h vs. 23 h
D
26 h vs. 17 h
log2FoldChange
log(baseMean)
Unchanged: 608
Up: 43
Down: 53
Unchanged: 624
Up: 45
Down: 35
Unchanged: 650
Up: 20
Down: 34
Unchanged: 535
Up: 72
Down: 97
E
17 h vs. 20 h
20 h vs. 23 h
23 h vs. 26 h
17 h vs. 26 h
20 h vs. 17 h
23 h vs. 20 h
26 h vs. 23 h
26 h vs. 17 h
Downregulated
Upregulated
LINE
LINE?
LTR
Other
Satellite
SINE
Unknown
F
MT2_Mm
MERVL-int
MT2_Mm
DESeq2 Normalized Counts
MT2_Mm
MERVL–int
***
NS.
NS.
***
***
NS.
Time point (h)
G
MERVL-target genes
(Macfarlan et al., 2012 (37);
Sakashita et al., 2023 (39); 85 genes)
17 h
20 h
23 h
26 h
z-score
≥ 2
1
0
-1
≤ -2
H
Differentially Expressed Transposons
(191 transposons)
17 h
20 h
23 h
26 h
z-score
≥ 2
1
0
−1
≤ −2
I
Cluster 1 (20 TEs)
MT2_Mm
B1_Mm
ORR1A3
Cluster 2 (26 TEs)
L1_Mus1
L1_Mm
RLTR16
Cluster 3 (22 TEs)
Lx3A
MTA_Mm
Cluster 4 (31 TEs)
ORR1A1
ORR1A2
IAPEz-int
MT2B2
Cluster 5 (35 TEs)
Cluster 6 (10 TEs)
Cluster 7 (21 TEs)
MERVL-int
ORR1A3-int
Cluster 8 (9 TEs)
Cluster 9 (17 TEs)
B1_Mus1
Expression Level
Time point (h)
Membership Score

Fig. S3. Transposable Element Dynamics in High-Resolution Mapping of Mouse Embryonic Genome Activation. A-D. DESeq2 analysis of transposable elements at the indicated time points using |Log2(FC)| > 0.5 and padj. < 0.01 for all comparisons. **E.** Pie chart showing the classification of differentially expressed transposable elements from panels A-D. **F.** DESeq2 normalized counts of MT2_Mm (left panel) and MERVL-int (right panel). A schematic of a full-length MERVL element is depicted in the upper panel. **G.** Heatmap of MERVL-target genes combined from *(37, 39)*, visualizing z-scores of log(CPM+1) gene counts. 85 genes displayed. **H.** Heatmap of differentially expressed transposable elements identified in panels A-D, visualizing z-scores of log(CPM+1) of transposon counts. 191 transposable elements visualized **I.** Fuzzy c-means clustering of median expression of differentially expressed transposons identified in panels A-D. The clustering has been performed using the package mfuzz with parameters c=9 and m=2. Representative transposons are indicated in the cluster panels.

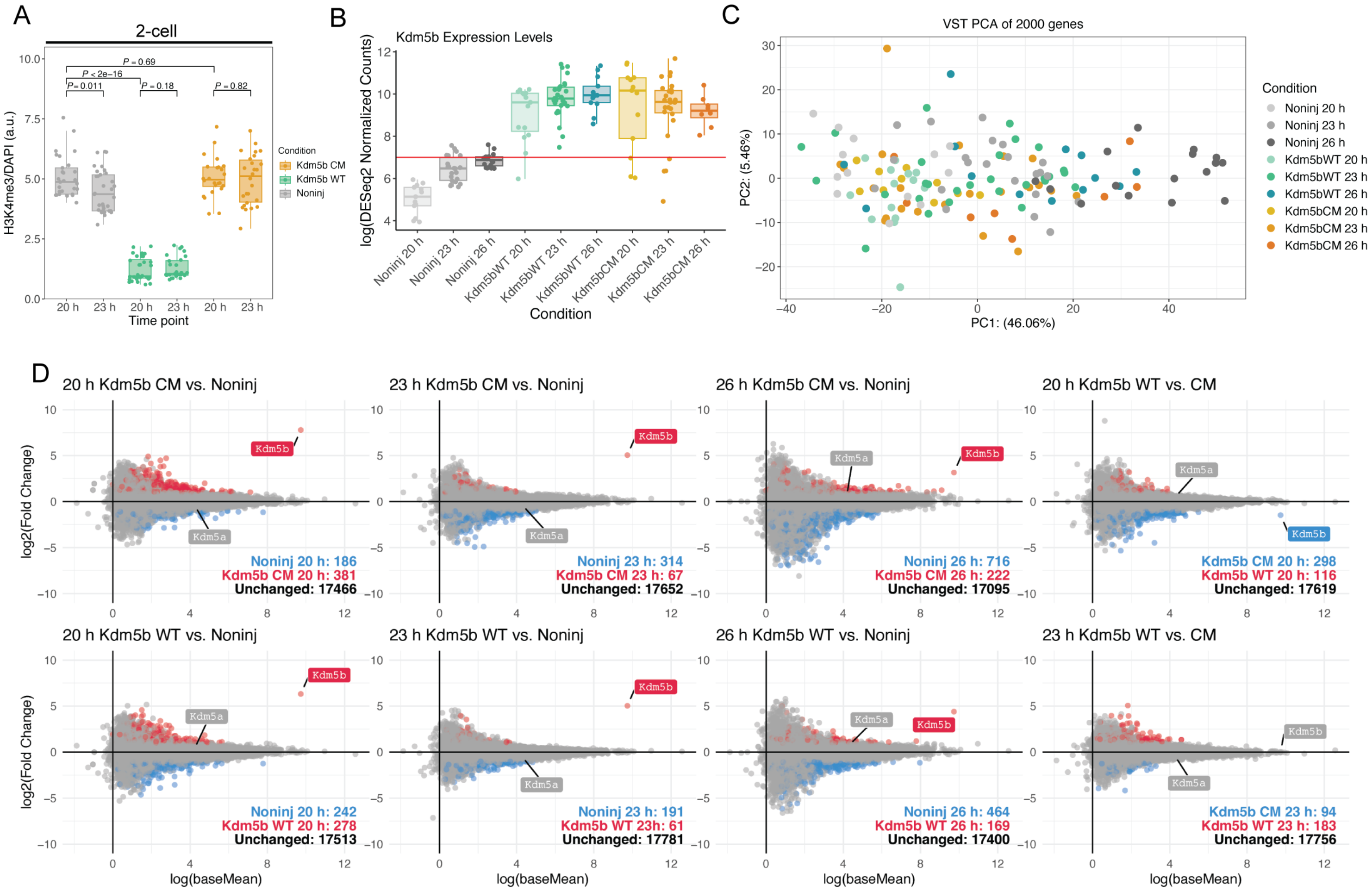

Fig. S4. Kdm5b Overexpression in Embryos Undergoing EGA. A. Quantification of the immunofluorescence data presented in (Fig. 4C). This analysis was performed on single optical z-slices where the nucleus was the widest. Mean intensities of H3K4me3 normalized top DAPI are shown. The samples were compared using Wilcoxon rank-sum tests**. B.** log(DESeq2 normalized counts) of Kdm5b transcript in single embryos. The red line indicates the retention threshold of samples for downstream analyses. In microinjected groups, samples with a Kdm5b expression level below the threshold of counts < log(7) were filtered out for downstream analysis. **C.** Principal Component Analysis of Kdm5b overexpressing embryos and Noninj controls over the top 2000 most variable genes following DESeq2 counts normalization and variance stabilizing transformation. **D.** Differentially expressed genes between indicated comparisons obtained with DESeq2 and parameters |Log2(FC)| > 1 and padj. < 0.05 for all comparisons. Legend labels indicate the number of genes enriched in each condition.

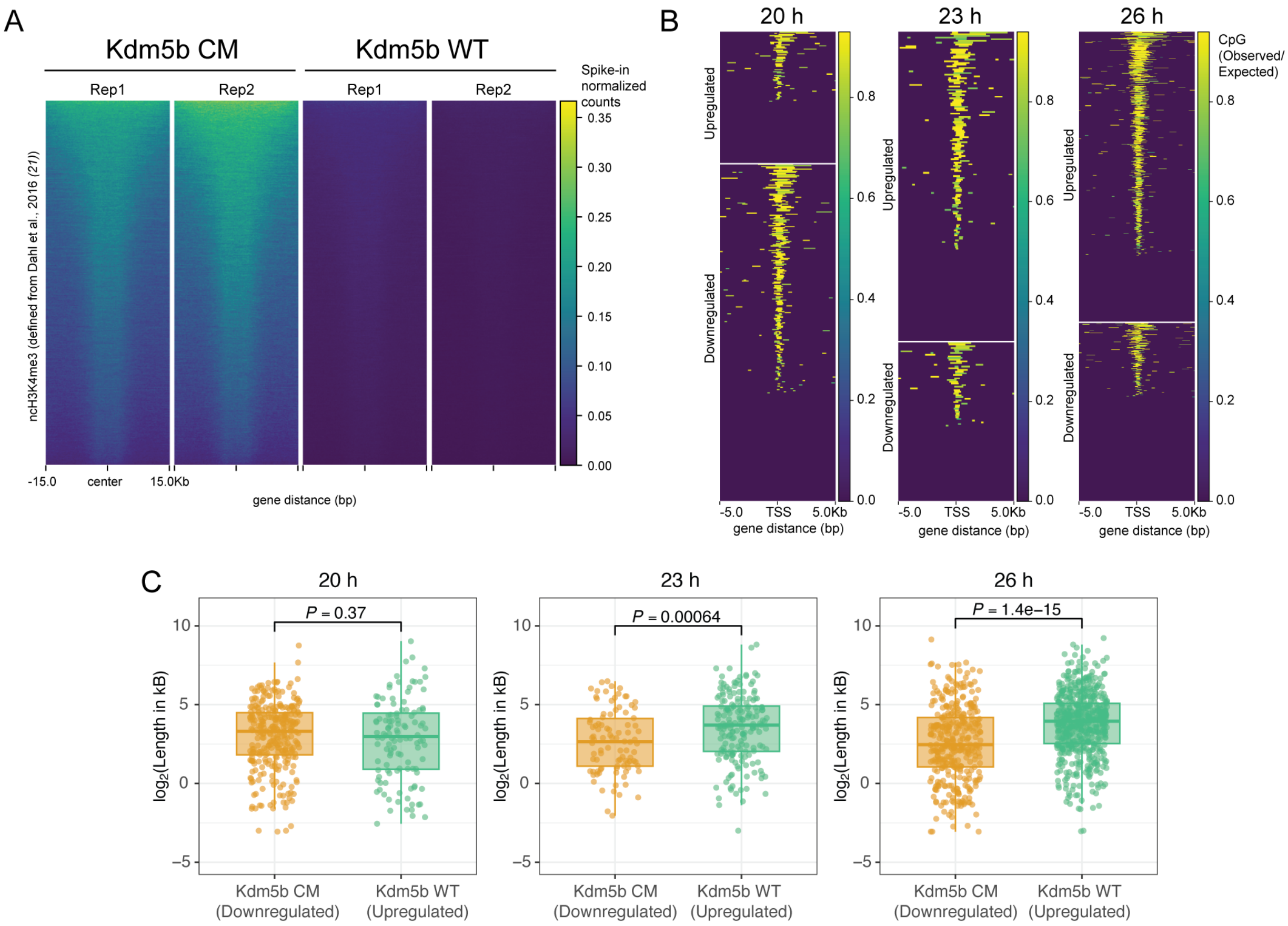

D

Rank	Motif	Name	P-value	log P-pvalue	q-value (Benjamini)	# Target Sequences with Motif	% of Targets Sequences with Motif	# Background Sequences with Motif	% of Background Sequences with Motif
1		Sp1(Zf)/Promoter/Homer	1e-32	-7.550e+01	0.0000	282.0	48.37%	19715.5	25.17%
2		KLF17(Zf)/2cell-Klf17-CutnTag(GSE211845)/Homer	1e-26	-6.207e+01	0.0000	357.0	61.23%	30467.1	38.89%
3		KLF3(Zf)/MEF-Klf3-ChIP-Seq(GSE44748)/Homer	1e-18	-4.343e+01	0.0000	324.0	55.57%	29099.0	37.15%
4		Sp5(Zf)/mES-Sp5.Flag-ChIP-Seq(GSE72989)/Homer	1e-17	-4.076e+01	0.0000	442.0	75.81%	45896.6	58.59%
5		Klf15(Zf)/Liver-Klf15-ChIP-Seq(GSE166083)/Homer	1e-15	-3.631e+01	0.0000	446.0	76.50%	47358.3	60.45%
6		KLF1(Zf)/HUDEP2-KLF1-CutnRun(GSE136251)/Homer	1e-15	-3.491e+01	0.0000	413.0	70.84%	42775.1	54.60%
7		Sp2(Zf)/HEK293-Sp2.eGFP-ChIP-Seq(Encode)/Homer	1e-11	-2.648e+01	0.0000	486.0	83.36%	55640.2	71.03%
8		Maz(Zf)/HepG2-Maz-ChIP-Seq(GSE31477)/Homer	1e-8	-1.969e+01	0.0000	460.0	78.90%	53234.0	67.95%
9		KLF14(Zf)/HEK293-KLF14.GFP-ChIP-Seq(GSE58341)/Homer	1e-8	-1.954e+01	0.0000	506.0	86.79%	60476.5	77.20%
10		Klf9(Zf)/GBM-Klf9-ChIP-Seq(GSE62211)/Homer	1e-7	-1.690e+01	0.0000	249.0	42.71%	25115.0	32.06%

E

Rank	Motif	Name	P-value	log P-pvalue	q-value (Benjamini)	# Target Sequences with Motif	% of Targets Sequences with Motif	# Background Sequences with Motif	% of Background Sequences with Motif
1		DUX(Homeobox)/C2C12-Dux-ChIP-Seq(GSE87279)/Homer	1e-7	-1.633e+01	0.0000	8.0	2.23%	132.5	0.15%
2		KLF17(Zf)/2cell-Klf17-CutnTag(GSE211845)/Homer	1e-6	-1.598e+01	0.0000	128.0	35.75%	21229.2	23.47%
3		Sp1(Zf)/Promoter/Homer	1e-6	-1.387e+01	0.0001	84.0	23.46%	12612.0	13.95%
4		KLF5(Zf)/LoVo-KLF5-ChIP-Seq(GSE49402)/Homer	1e-5	-1.275e+01	0.0003	234.0	65.36%	48282.8	53.39%
5		Klf15(Zf)/Liver-Klf15-ChIP-Seq(GSE166083)/Homer	1e-4	-1.137e+01	0.0011	202.0	56.42%	40804.0	45.12%
6		Sp5(Zf)/mES-Sp5.Flag-ChIP-Seq(GSE72989)/Homer	1e-4	-1.107e+01	0.0012	192.0	53.63%	38466.1	42.53%
7		Elk1(ETS)/Hela-Elk1-ChIP-Seq(GSE31477)/Homer	1e-4	-1.059e+01	0.0017	128.0	35.75%	23441.3	25.92%
8		KLF1(Zf)/HUDEP2-KLF1-CutnRun(GSE136251)/Homer	1e-4	-1.016e+01	0.0023	180.0	50.28%	36001.4	39.81%
9		Zfp541(Zf)/Sperm-Zfp541-ChIP-Seq(GSE163916)/Homer	1e-4	-9.940e+00	0.0025	143.0	39.94%	27240.7	30.12%
10		KLF3(Zf)/MEF-Klf3-ChIP-Seq(GSE44748)/Homer	1e-4	-9.504e+00	0.0035	125.0	34.92%	23281.5	25.74%

Fig. S5. H3K4me3 Signal over ncH3K4me3 Domains and Sequence features of Kdm5b Differentially Expressed Genes. A. H3K4me3 signal over ncH3K4me3 domains that have been defined from *(21)*. The signal visualized is from a public CUT&Tag dataset from (*45*). The data was generated in early 2-cell embryos upon overexpression of Kdm5b WT or Kdm5b CM. *D. melanogaster* spike-in normalized counts are visualized in ±15 kB regions relative to the center of each domain. **B.** CpG density over TSSs (±5 kB) of differentially expressed genes when comparing Kdm5b WT (upregulated) vs. Kdm5b CM (downregulated) at 20 h (left panel), 23 h (middle panel), and 26 h (right panel). The Observed/Expected ratios of CpG islands from USCS Table Browser for the mm10 genome are visualized. **C.** Gene lengths of differentially expressed genes when comparing Kdm5b WT (upregulated) vs. Kdm5b CM (downregulated) at 20 h (left panel), 23 h (middle panel), and 26 h (right panel). The groups are compared using Wilcoxon rank sum tests and the *P*-values are indicated. **D-E.** Top 10 transcription factor motifs identified using HOMER (known motifs) across promoters (1000 bp upstream of TSS and 200 bp downstream of TSS) of differentially expressed genes when comparing Kdm5b WT vs. Kdm5b CM at 26 h. Results of promoters of upregulated (Kdm5b WT) genes are visualized in panel (**D**) and for downregulated (Kdm5b CM) genes, the results are visualized in panel (**E**).

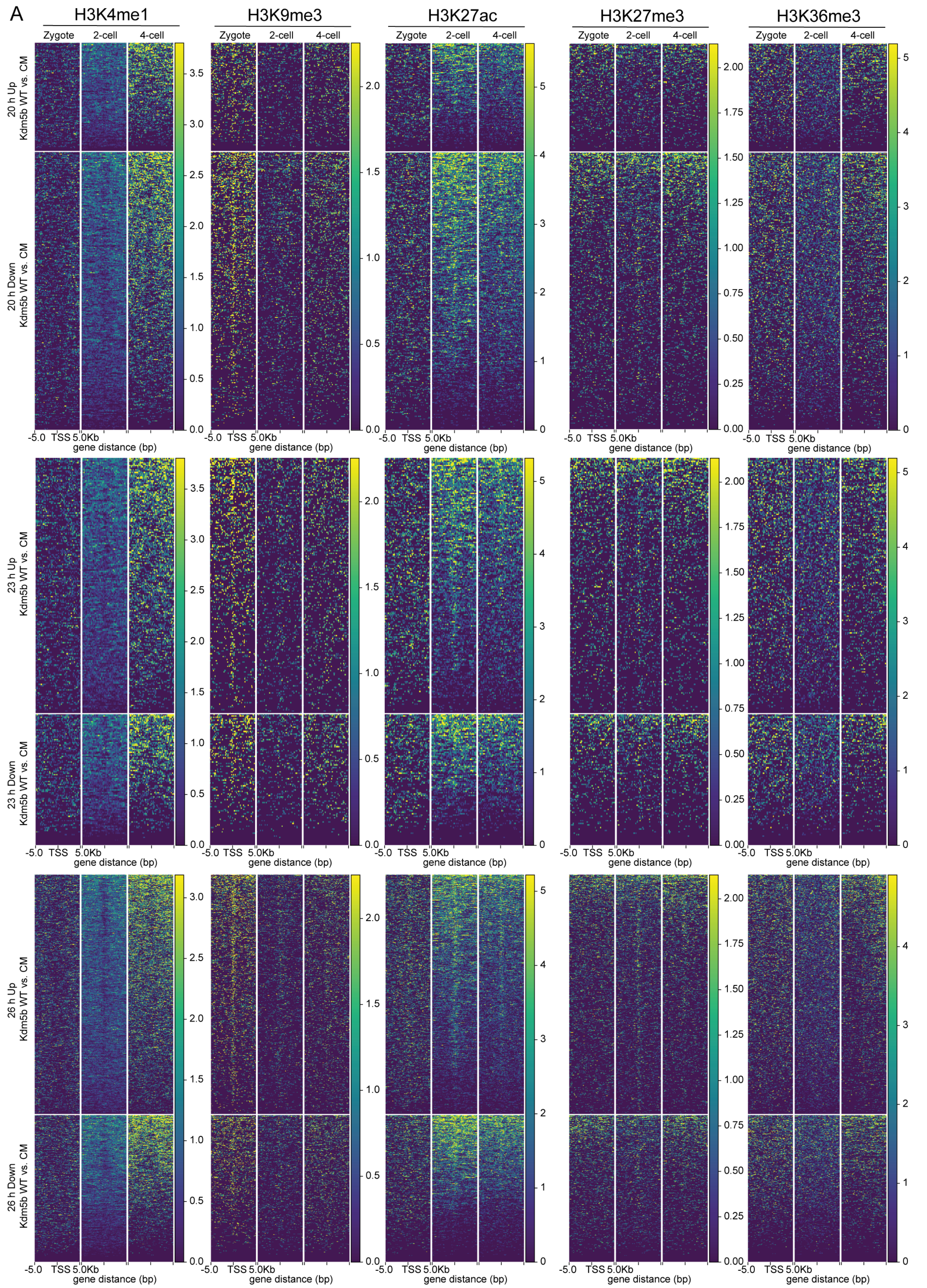

Fig S6. Histone PTM occupancy across TSS regions of Differentially Expressed Genes in Kdm5b Overexpressing Embryos. A. Visualization of TSS enrichment of H3K4me1, H3K9me3, H3K27ac, H3K27me3, and H3K36me3 using public TACIT data (*47*) from zygotes, 2-cell and 4-cell stage embryos across differentially expressed genes from Kdm5b WT (Up) vs. Kdm5b CM (Down) comparisons at 20 h (top row), 23 h (middle row), and 26 h (bottom row). Regions ±5 kB relative to the TSS are visualized.

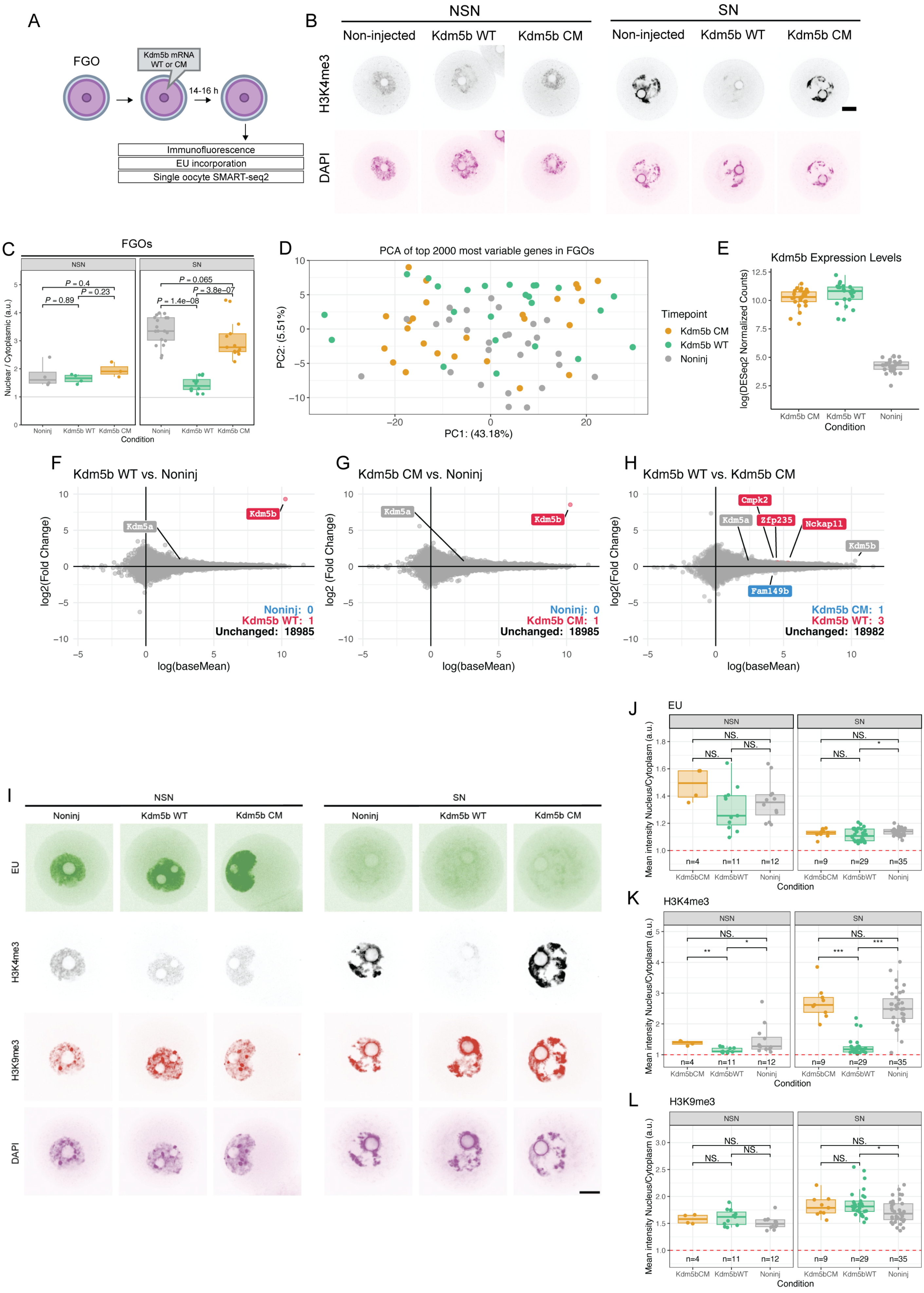

A
FGO
Kdm5b mRNA WT or CM
14-16 h
Immunofluorescence
EU incorporation
Single oocyte SMART-seq2
B
NSN
SN
Non-injected
Kdm5b WT
Kdm5b CM
H3K4me3
DAPI
C
FGOs
NSN
SN
P = 0.4
P = 0.23
P = 0.89
P = 0.065
P = 3.8e−07
P = 1.4e−08
Nuclear / Cytoplasmic (a.u.)
Noninj
Kdm5b WT
Kdm5b CM
Condition
D
PCA of top 2000 most variable genes in FGOs
PC1: (43.18%)
PC2: (5.51%)
Timepoint
Kdm5b CM
Kdm5b WT
Noninj
E
Kdm5b Expression Levels
log(DESeq2 Normalized Counts)
Condition
F
Kdm5b WT vs. Noninj
Kdm5b
Kdm5a
Noninj: 0
Kdm5b WT: 1
Unchanged: 18985
log2(Fold Change)
log(baseMean)
G
Kdm5b CM vs. Noninj
Kdm5a
Kdm5b
Noninj: 0
Kdm5b CM: 1
Unchanged: 18985
H
Kdm5b WT vs. Kdm5b CM
Cmpk2
Kdm5a
Zfp235
Nckap11
Kdm5b
Fam149b
Kdm5b CM: 1
Kdm5b WT: 3
Unchanged: 18982
I
NSN
SN
Noninj
Kdm5b WT
Kdm5b CM
EU
H3K4me3
H3K9me3
DAPI
J
EU
Mean intensity Nucleus/Cytoplasm (a.u.)
NS.
n=4
n=11
n=12
n=9
n=29
n=35
Kdm5bCM
Kdm5bWT
Noninj
Condition
K
H3K4me3
L
H3K9me3

Fig. S7. Kdm5b Overexpression in Fully Grown Oocytes. A. Schematic of FGO collection and microinjection with Kdm5b WT or CM. **B.** Representative immunofluorescence images of FGOs following Kdm5b overexpression, split by nuclear classification: NSN and SN. Single z-slices are displayed. The scale bar is 20 μm. **C.** Quantification of the data presented in panel B. The groups were compared using Wilcoxon rank sum tests. N = 2 collections, n = 55 oocytes quantified. The quantifications were done on single slices, where the nucleus was most visible. **D.** Principal Component Analysis of individual oocyte transcriptomes after Kdm5b overexpression and SMART-seq2, as well as Noninj controls. The PCA is based on the top 2000 most variable genes following DESeq2 counts normalization and variance stabilizing transformation. N = 4 collections, n = 76 oocytes. **E.** log(DESeq2 normalized counts) of Kdm5b levels in sequenced oocytes according to condition. **F-H.** Differentially expressed genes of the indicated FGO conditions obtained using DESeq2 parameters $|Log2(FC)| > 0.5$ and padj. < 0.05 for all comparisons. Legend labels indicate the number of genes enriched in each condition. **I.** Representative EU incorporation and immunofluorescence images of FGOs following Kdm5b overexpression, split by nuclear classification and microinjection condition. EU incorporation is displayed in green (top row). The oocytes have been co-stained with antibodies against H3K4me3 (grey signal, second row) and H3K9me3 (red signal, third row). Single z-slices are displayed. The scale bar is 20 μm. **J-L.** Quantifications of the images presented in (I). Nuclear-to-cytoplasm ratio has been calculated across the sum projections of the acquired z-stacks for each channel: EU (J), H3K4me3 (K), and H3K9me3 (L). The groups were compared using Student's T-tests. The significance is indicated as * ($P<0.05$), ** ($P<0.01$), *** ($P<0.001$), and NS. (not significant).

Supplemental Tables 1-5.

Table S1. Gene lists derived from c-means clustering analysis of the EGA time course dataset (related to Fig. 2F).

Table S2. Gene Ontology (GO) enrichment analysis of gene sets associated with discrete clusters identified by c-means clustering (related to Fig. 2F and Table S1).

Table S3. Differentially Expressed Genes (DEGs) identified through sequential and end-point DESeq2 pairwise comparisons of the EGA time course transcriptome data (related to Fig. 3A–D).

Table S4. Differentially Expressed Genes (DEGs) identified at defined time points of the 2-cell stage upon overexpression of Kdm5b wild-type (WT) or catalytically inactive mutant (CM) (related to Fig. 4F).

Table S5. Differentially Expressed Genes (DEGs) identified by comparative analysis of embryo RNA samples subjected to experimental polyadenylation versus non-polyadenylated controls, at indicated 2-cell stage time points (related to Fig. S2).
